# The Impact of Response Instruction and Target Group on the BIAS Map

**DOI:** 10.3389/fpsyg.2020.566725

**Published:** 2020-10-06

**Authors:** Andrej Findor, Barbara Lášticová, Matej Hruška, Miroslav Popper, Luca Váradi

**Affiliations:** ^1^Faculty of Social and Economic Sciences, Comenius University, Bratislava, Slovakia; ^2^Institute for Research in Social Communication, Slovak Academy of Sciences, Bratislava, Slovakia; ^3^Nationalism Studies Program, Central European University, Budapest, Hungary; ^4^Faculty of Social Science, Eötvös Loránd University, Budapest, Hungary

**Keywords:** BIAS map, Stereotype Content Model, response instruction, target group, the Roma, the Hungarians

## Abstract

Response instructions—inviting participants to respond from a certain perspective—can significantly influence the performance and construct validity of psychological measures. Stereotype Content Model (SCM) and then the BIAS map (“behaviors from intergroup affect and stereotypes”) were originally developed as universal measures of shared cultural stereotypes—participants’ perceptions of what most of the people in a society think about the target group—and their related social-structural antecedents, emotions and behavioral tendencies. Yet a number of studies have adopted a different response instruction focusing on individual stereotypes—what the participants personally think about the target group. So far, there is little evidence to suggest how these two different response instructions (individual vs. shared cultural perspective) might influence the performance of the BIAS map, especially when applied to target groups that elicit different normative and social desirability concerns. To provide novel evidence, we conducted an experiment with a representative sample of ethnic Slovaks (*N* = 1269). In a 2 × 2 factorial design, we found response instruction (individual vs. shared cultural perspective) and target group [stigmatized ethnic minority (the Roma) vs. non-stigmatized ethnic minority (the Hungarians)] had significant effects on the BIAS map and their interaction had significant effects on the social structure and behavioral tendencies (but not on stereotypes and emotions) scales. Exploratory analysis also points to partial influence on the mediation hypothesis underlying the BIAS map and minor effects on its scale properties. Our evidence suggests that the difference between individual stereotypes and shared cultural stereotypes partially depends on the target group in question and that they should be treated as two potentially separate constructs.

## Introduction

Response instructions—asking participants to answer from a certain perspective—can have a significant impact on the performance and construct validity of psychological measures (Ployhart and Ehrhart, 2003; Pauls and Crost, 2005; McDaniel et al., 2007). The Stereotype Content Model (SCM) and the BIAS (“behaviors from intergroup affect and stereotypes”) map were originally devised to assess stereotypes from a shared cultural perspective—participants’ perceptions of what most of the people in their society think about the target group (Fiske et al., 2002; Cuddy et al., 2007; Cuddy et al., 2008). However, many subsequent studies utilizing the SCM and the BIAS map instructed participants to respond from their individual perspective—what they personally think about the target group. Despite recent concerns about this practice (Bye and Herrebrøden, 2018; Kotzur et al., 2019a) and emerging evidence about the impact of response instruction format on the warmth and competence scales of the SCM (Popper and Kollárová, 2018; Kotzur et al., 2020), little is known about whether instructions inviting responses from individual and shared cultural perspectives influence the BIAS map (including the SCM), especially when applied to target groups that elicit different normative and social desirability concerns, as in Slovakia’s intergroup relations context (the Roma—a stigmatized ethnic minority vs. the Hungarians—a non-stigmatized ethnic minority). In order to fill this gap, we seek to provide novel evidence by testing the hypotheses about the impact of response instruction, target group, and their interaction on the BIAS map scores in a factorial between-subject experiment. In addition, we also explore the potential impact of these two factors on scale properties and the mediation hypothesis underlying the BIAS map.

### The SCM and the BIAS Map

The SCM (Fiske et al., 2002) has become a universal measure of intergroup perception, describing the content and social-structural antecedents of stereotypical beliefs about diverse categories of people (e.g., societal subgroups based on their gender, ethnicity, or sexual orientation) across America, Europe and Asia (Cuddy et al., 2009; Fiske, 2018). It posits that the perceived socio-economic status and competitiveness of out-group members predict how in-group members evaluate the out-group members along two universal dimensions of social cognition—competence and warmth—which elicit the corresponding affective reactions of admiration, envy, pity and contempt (Fiske et al., 2002; Fiske et al., 2007; Caprariello et al., 2009). The SCM was subsequently developed into a BIAS map framework to include emotions as well as components of behavioral tendencies (Cuddy et al., 2007, 2008). The BIAS map framework integrated the SCM’s composite scales—social structure scale (status and competitiveness subscales), stereotypes scale (competence and warmth subscales), emotions scale (contempt, admiration, pity and envy subscales)—with the behavioral tendencies scale (active facilitation, active harm, passive facilitation and passive harm subscales) (Cuddy et al., 2007, 2008). Central to the BIAS map model is the “mediation hypothesis”: that the emotional reactions of admiration, envy, pity and contempt mediate the relationship between warmth stereotypes and the behavioral tendencies of active harm (harassing) or active facilitation (helping) and competence stereotypes and the behavioral tendencies of passive harm (neglecting) or passive facilitation (associating). According to the mediation hypothesis underlying the BIAS map (Cuddy et al., 2007), admired target groups perceived as warm and competent evoke both active and passive facilitation tendencies; hated groups perceived as cold and incompetent elicit both active and passive harm tendencies; envied groups perceived as cold and competent prompt passive facilitation and active harm tendencies; and pitied groups perceived as warm and incompetent evoke active facilitation and passive harm tendencies.

### Response Instructions in the SCM and BIAS Map

The SCM and the BIAS map instruments adopted identical response instructions that, rather than asking participants about evaluations of target groups from their own *individual perspective*, tapped into their *perceptions* of these evaluations from *a shared cultural perspective*, arguably safeguarding their responses against social desirability bias (Fiske et al., 2002). Originally, both instruments used the group-centered understanding of stereotypes—“beliefs about the predominant cultural view of a group” rather than the individual-centered one—“personal beliefs about the characteristics of a group” (Krueger, 1996, p. 536). In the initial SCM study, “participants were instructed to make the ratings, using 5-point scales (1 not at all to 5 extremely), on the basis of how the groups are viewed by American society. The instruction was, “We are not interested in your personal beliefs, but in how you think they are viewed by others.” As in all our studies, this instruction was intended to reduce social desirability concerns and to tap perceived cultural stereotypes” (Fiske et al., 2002, pp. 884–885). This original response instruction, used in the SCM and the BIAS map to investigate perceptions of stereotypes from *a shared cultural perspective*, has been employed in numerous observational and experimental studies asking participants to view the target groups or categories of people through the eyes of “most of the people” in their country or “others in the society,” or to consider them in terms of how they are “viewed by the … society” or “people like you” (Cuddy et al., 2007; Asbrock, 2010; Cichocka et al., 2015; Bye and Herrebrøden, 2018, Study 1; Cuddy et al., 2009, Study 1; Eckes, 2002; Koenig and Eagly, 2014; Janssens et al., 2015; Froehlich and Schulte, 2019; Grigoryan et al., 2019; Studies 1a, 1b, and 1c; Lee and Fiske, 2006; Sadler et al., 2015; Stanciu, 2015; Stanciu et al., 2017; Kotzur et al., 2019a).

Nonetheless, a number of studies employing the SCM and the BIAS map have used a different response instruction, focusing on participants’ evaluations from their own *individual perspective*. Diverging from the original social, group-centered, shared cultural perspective, these studies instructed their participants to express personal stereotypical beliefs, by for instance asking them about “your opinion about a particular group” or “how (e.g., warm) do you think this person is” (Becker and Asbrock, 2012; Koschate et al., 2012; Matthews and Levin, 2012; Durante et al., 2014; Awale et al., 2018; Constantin and Cuadrado, 2019; Kotzur et al., 2019b, Study 2; Sweetman et al., 2013; Ponsi et al., 2016; Sink et al., 2018, Study 2; Ufkes et al., 2012).

### Personal Beliefs and Social Norm Perceptions

An abundant evidence in the social psychology literature points to the discrepancy between what people personally think and their perceptions of social norms: what they perceive others think about an issue (Tankard and Paluck, 2016). Pluralistic ignorance occurs when people falsely estimate the majority attitude to be different from their own (Katz et al., 1931; Prentice and Miller, 1993; Van Boven, 2000) and has been defined as “shared false ideas” by Shamir and Shamir (1997). It can take the form of unawareness, when people believe that everyone else has the same or a different opinion from theirs, or minor bias (Shamir and Shamir, 1997). In relation to intergroup attitudes, false perception of the majority view was often found to follow a typical pattern: people were more open when asked about their own views than when asked about their perceptions of attitudes within their social environment or among the population at large. This special type of pluralistic ignorance, which is typically associated with overestimations of the acceptance of prejudice in society, is called *conservative bias* (Fields and Schuman, 1976).

The relationship between social norm perceptions and individual intergroup attitudes and behavior has also been studied beyond pluralistic ignorance or conservative bias, e.g., Sherif and Sherif’s (1953) Group Norm Theory. Crandall et al. (2002) found that people closely follow perceived norms (what other people do and ought to do) when expressing prejudice and also adjust their intended behavior to what they perceive to be acceptable in their in-groups. Moreover, a number of experimental studies have demonstrated that the perceived social consensus (prevalent opinions of other relevant people) regarding the target groups has a validating effect on individuals’ personal attitudes, stereotypic beliefs and behaviors toward these target groups (Haslam et al., 1996; Wittenbrink and Henly, 1996; Sechrist and Stangor, 2001; Stangor et al., 2001a,b). This line of research led to the decision to ask about perceptions of others’ stereotypical beliefs rather than about the participant’s personal stereotypical beliefs in the SCM and BIAS map, in an attempt to “reduce social desirability concerns” (Fiske et al., 2002, pp. 884–885). After all, social desirability bias—“the tendency of research subjects to choose responses they believe are more socially desirable or acceptable rather than choosing responses that are reflective of their true thoughts or feelings” (Grimm, 2010)—stems from the social norms that indicate which attitudes, beliefs or behaviors are perceived as socially acceptable or desirable in the given social context or situation (Nederhof, 1985).

The validating influence of perceived normative consensus and related social desirability concerns suggest a potential convergence between the expression of stereotypes and prejudice from personal and social normative perspectives. However, previous research suggests that it would not apply equally to all target groups (Crandall et al., 2002; Crandall and Eshleman, 2003).

### The Impact of Response Instruction on the BIAS Map

Although the relationship between personal beliefs and attitudes on the one hand and perceptions of others’ beliefs and attitudes on the other has been extensively described from various theoretical perspectives, there is still a limited empirical evidence on how response instructions prompting an individual vs. shared cultural perspective might influence the performance of the BIAS map measure.

This inconsistency in the use of response instructions in the SCM and the BIAS map and the potential repercussions for the performance and properties of the two measures was highlighted by Kotzur et al. (2019a), who argue for the systematic evaluation of the potential impact of using individual vs. shared cultural perspective response instructions on the SCM. Similarly, Bye and Herrebrøden (2018) assert that the impact of individual vs. shared cultural perspective response instructions on the BIAS map deserves closer scrutiny, especially since this may be one of the factors responsible for the mixed empirical support for the mediation hypothesis proposed by the BIAS map framework.

Emerging evidence suggests that these different response instructions influence the level of reported stereotypes. In cognitive interviews conducted with a convenience sample of secondary school students and adults in Slovakia (*N* = 24), Popper and Kollárová (2018) found that participants expressed more negative stereotypes about the Roma when they were instructed to answer from the viewpoint of the majority of people in Slovakia than when they were asked to respond from the perspective of people who they are close to or from their own personal perspective. Participants reported that they found responding from their own personal perspective more agreeable and less difficult than responding from the other two perspectives. However, the small number of participants make these findings difficult to generalize. Recently, Kotzur et al. (2020) observed that German participants gave less positive assessment of multiple groups “but only on already depreciated stereotype content dimensions” when instructed to respond from the societal perspective compared to the individual perspective instruction. Moreover, they have argued that the mean level differences in reported stereotypes between different responses instructions might not under all circumstances reflect the relative position of different target groups within the two-dimensional stereotype content space (Kotzur et al., 2020). Even small differences in the mean level of reported stereotypes can be indicative of the distinctive social perceptions and behaviors toward members of different target groups, with some groups (including the Roma) being outliers within their particular SCM quadrant (see e.g., Grigoryev et al., 2019).

Kotzur et al. (2020) recognized the limited scope of their analysis focusing solely on stereotypes scales (warmth and competence) of the SCM and suggested that future research should also investigate other components of the SCM and its extensions (the BIAS map). To answer their call, we seek to extend their evidence to include the potential effects of response instruction, the target group, and their interaction on the performance and properties of the social structure, stereotypes, emotions and behavioral tendencies scales, and the mediation hypothesis of the BIAS map.

### The Impact of Target Group on the BIAS Map

The kind of target group being studied may also feed into the effects of the individual vs. shared cultural perspective instructions on participants’ responses to the BIAS map. Different target groups are associated with different normative, and more specifically, social desirability effects on participants’ reluctance to express stereotypes and prejudice in self-reported measures. As Crandall et al. (2002) point out, due to the perceived normative consensus, hostility and prejudice against certain target groups is normatively more sanctioned than against other target groups. Prejudice against rapists and child abusers is more justified and its expression is suppressed less than hostility against the elderly and people with hearing loss. Often, it is not even considered prejudice. Social conformity with perceived majority beliefs and attitudes can lead to suppression and under-reporting of forms of prejudice that attract normative disapproval (Crandall and Eshleman, 2003). Perceptions of the majority’s view of whether individuals will express stereotypes, prejudices and discrimination therefore depend on the specific target group being investigated. In psychological measures that rely on self-reports, different target groups will attract different normative acceptability and social desirability concerns.

The presumed impact of the target group on the expression of individual stereotypes and perceptions of shared cultural stereotypes is well illustrated by comparing two largest ethnic minorities in Slovakia—Roma and Hungarians. These groups are not commonly studied in the SCM and the BIAS map scholarship. When compared with the Roma, the Hungarian ethnic minority in Slovakia enjoys a higher status, which is reflected in their standard of living that is similar to that of the Slovak majority; in the extensive system of schools with Hungarian language instruction; well-organized Hungarian ethnic political parties that have repeatedly formed part of governing coalitions; and the vigorous political, economic and cultural support of their kin-state—Hungary (Stroschein, 2018). In contrast, the Roma communities in Slovakia suffer from extreme poverty, social exclusion, and spatial segregation (Rochovská and Rusnáková, 2018). They are also subject to stigmatization, marginalization, blatant prejudice and dehumanization (Kteily et al., 2015, Study 4). Evidence suggests that anti-Gypsyism remains “the last acceptable prejudice in Europe” (Kende et al., 2020). Kende et al. (2017) maintain that the normative climate in Slovakia (and Hungary) encourages the expression of anti-Roma prejudice. They consider the hostility against the Roma to be “one of the most severe forms of bias all over Europe” that reflects “socially approved dominant societal norms” (p. 12). Similarly, Cichocka et al. (2015) claim that members of the Roma minority in Poland “are least protected by ‘political correctness’ norms and are the most frequent target of hate speech in Poland” (p. 796).

However, considering the importance of the cultural and societal context for understanding intergroup relations (Pettigrew, 2018), the role of context must be accounted for when studying the effect of the target group on the individual and shared cultural stereotypes. As Bilewicz (2012) observes, the same ethnic minority group (e.g., the Roma) can be “subtly infra-humanized in Britain” and “still harshly and openly dehumanized in Romania” (p. 428). The same target group can elicit different social desirability concerns engendered by specific cultural and societal intergroup contexts and normative climates. The presumed effect of the target group on the BIAS map is thus category- and context-sensitive in equal measure (Grigoryan et al., 2019).

### The Present Research

So far, the design of previous studies on the SCM and the BIAS map makes it difficult to assess the impact of the target group on the performance and properties of these scales. In four studies in Fiske et al. (2002) and two studies in Cuddy et al. (2007) participants rated between 4 and 25 groups simultaneously (in Study 2 of Fiske et al., 2002, the rated groups were split in half and presented in a reversed order). Similarly, in three studies in Kotzur et al. (2020) participants assessed between 6 and 38 groups at once. Since these articles report no random order of the rated groups, their design could allow for the effects of question order on participants’ responses due to social comparison and “norm of reciprocity or fairness” (Hyman and Sheatsley, 1950; Oldendick, 2008). Random ordering of scale presentation in these studies could have overcome these potential limitations (Perreault, 1975). To control for these potential effects of question order and explore the impact of the target group, we adopted an experimental design in which participants rate one target group on all dimensions of the BIAS map measure. Following the advice of Crosby et al. (1980) and Crandall et al. (2002) that experiments (compared to surveys) are less obtrusive measures of prejudice (and stereotypes) that better account for social conformity pressures, we chose not to adopt a survey design in which all participants would answer the BIAS measure in all response instruction and target group conditions. Instead, in line with recommendations of Bu and Borgida (2020), we opted for an experimental 2 × 2 factorial design that would allow us to test the anticipated interaction between the effects of response instruction and target group on participants’ responses to the BIAS map.

In the present study, we experimentally test the hypothesized impact of the response instruction (individual perspective vs. shared cultural perspective), target group [stigmatized out-group in Slovakia (Roma) vs. non-stigmatized out-group in Slovakia (Hungarian)], and their interaction on the BIAS map scores. Based on the literature (Fields and Schuman, 1976) and previous findings (Popper and Kollárová, 2018; Kotzur et al., 2020), we test the following hypotheses:

H1 (Response instruction effect): Participants instructed to respond from a shared cultural perspective will report less favorable evaluations in the BIAS map scales than participants instructed to respond from their individual perspective.

H2 (Target group effect): Participants instructed to respond about stigmatized target group (Roma) will report less favorable evaluations in the BIAS map scales than participants responding about non-stigmatized target group (Hungarian).

H3 (Interaction effect): Target group interacts with response instruction to influence BIAS map scores such that stigmatized target group (Roma) elicits less favorable evaluations in the BIAS map scales when using a shared cultural perspective (compared to individual perspective) than non-stigmatized target group (Hungarian).

We also explore the potential impact of response instruction and target group on scale properties (skewness and kurtosis of BIAS map subscales, multivariate skewness and kurtosis of BIAS map scales, reliability, scalability) and the mediation hypothesis underlying the BIAS map.

## Materials and Methods

### Participants

Data were collected in October 2017 from 1,393 participants to obtain a quota-representative (gender, age, education, region, and population size of the municipality) sample of a general Slovak population. 21 participants were excluded for exceeding quotas and 103 for failing attention checks (22 participants from the “Roma + shared cultural perspective” condition, 40 from the “Hungarian + shared cultural perspective” condition, 23 from “Roma + individual perspective” condition, 18 from “Hungarian + individual perspective” condition). The final sample comprised 1,269 ethnic Slovak participants (647 women—50.1%; aged 18–65 years, *M* = 39.6, *SD* = 13.22), whose gender, age, education and region of residence were representative of the general Slovak population. Sample size was determined *a priori* by rule of thumb: a minimum of 300 participants in each condition; hence we expected at least 1,200 valid responses. A *post hoc* sensitivity analysis for fixed, special, main effects and interactions in ANOVA using G^∗^Power with *α* = 0.05, numerator df = 1 and four groups showed that we had an 80% chance of detecting a main effect as small as *f* = 0.08 (*d* = 0.16). Participants were recruited from a national online panel administered by 2muse agency and received points for completing the questionnaires that could be exchanged for various rewards.

### Materials and Procedure

The adaptation and validation of the Slovak version of the BIAS map (Lášticová et al., underv review) was based upon Fiske et al. (2002, Study 1) and Cuddy et al. (2007). Participants were randomly allocated to one of the four conditions in the 2 × 2 factorial design (individual perspective vs. shared cultural perspective) and [the Roma (stigmatized, low status out-group in Slovakia) vs. Hungarians (non-stigmatized, high status out-group in Slovakia)]. In each condition they were instructed to answer on a scale from 1 (*not at all*) to 5 (*extremely*) reflecting how they personally viewed, felt and would behave (individual perspective) or how they thought most people in Slovakia would view, feel or behave (shared cultural perspective) toward the Roma or Hungarians. All participants answered the stereotypes scale [*competence* subscale (competent, capable, skilful), *warmth* subscale (warm, good-natured, friendly)], social structure scale [*status* subscale (living standard, prestigious jobs, social status), *competitiveness* subscale (special breaks, resources, power)], emotions scale [*contempt* subscale (contempt, disgust), *admiration* subscale (admire, proud), *pity* subscale (pity, sympathy), *envy* subscale (envious, jealous)] and behavioral tendencies scale [*active facilitation* subscale (help, protect), *active harm* subscale (fight, attack), *passive facilitation* subscale (cooperate with, associate with), *passive harm* subscale (exclude, demean)]. Participants also answered 10 questions about their motivation to express prejudice (Forscher et al., 2015) and 10 questions assessing their internal and external motivation to respond without prejudice (Plant and Devine, 1998). Subsequently, participants answered 12 questions regarding the quality and quantity of any direct contact they had with members of the target groups (“How often do you come into contact with the Roma/Hungarians? How often do you spend time with the Roma/Hungarians?”) and its valence (“How do you feel while doing so?”); extended contact (“How many friends do you have that you know have Romani/Hungarian friends?”); vicarious mass-mediated contact (“How often do you come across media reports about the Roma/Hungarians?”) and its valence (“What is the tone of these reports?”)^1^. Finally, participants answered socio-demographic questions about their education (only if these data had not been recorded in the online panel), political right–left self-classification, conservative–liberal self-identification on cultural and ethical issues, voting preferences, religion, frequency of attendance of religious services and social status. Due to the large number of items in the questionnaire, participants also answered two attention check questions. Those who provided incorrect answers were automatically excluded from the analysis.

### Statistical Analyses

We used Cronbach’s *α*, McDonald’s *ω* (for subscales consisting of at least three items, Tables 3–5 reported in Supplementary Material) and Mokken scale analysis (coefficient *H*) to assess the properties of the subscales of the BIAS map. Mokken scale analysis is used to investigate psychometric properties of a scale, comparing its actual Guttman errors to expected errors (resulting in scalability score) and assessing “whether each item evaluates the same underlying concept” (Park et al., 2019). When assumptions are violated, the omega coefficient provides a better assessment of the internal consistency (reliability) of a scale than the alpha coefficient does (Dunn et al., 2014). For subscales consisting of two items, we also report Spearman-Brown coefficients (Eisinga et al., 2013). In the Mokken scale analysis (MSA), based on non-parametric item response theory models, we first partitioned the variables into subscales using automated item selection procedure (AISP) and then calculated goodness-of-fit for each of the subscales (Andries van der Ar, 2012). A coefficient *H* above 0.5 indicates a scale with strong scalability; between 0.4 and 0.5 moderate; between 0.3 and 0.4 weak; and below 0.3 unsatisfactory scalability (Andries van der Ar, 2012).

To analyze the main effect of instruction and target group and their possible interaction we used robust non-parametric analysis of multivariate outcomes in factorial experiments via MANOVA.RM package (Friedrich et al., 2019), which allows for MANOVA-like test, but without assuming multivariate normality. Non-parametric tests are more suitable for data that violate assumptions of normality and equal covariances structure, and also perform better for small to medium samples (Arboretti et al., 2018). To account for the number of tests performed on non-independent data, the 5% threshold alpha for interaction and main effect tests was corrected using the M_eff_ method (Derringer, 2018). Using the meff function provided by Derringer (2018) we estimated a corrected effective number of tests for the set of 12 BIAS map subscales (M_eff_ = 10.39). The standard α threshold of 0.05 was then divided by M_eff_ to obtain the level of corrected α = 0.0048.

To test the mediation hypotheses, we computed four parallel multiple mediator models using the *mediate* function from the psych package (Revelle, 2019). To evaluate the presence or absence of a mediating relationship, we used bootstrapped (10,000 samples) indirect effects.

## Results

**FIGURE 1:**
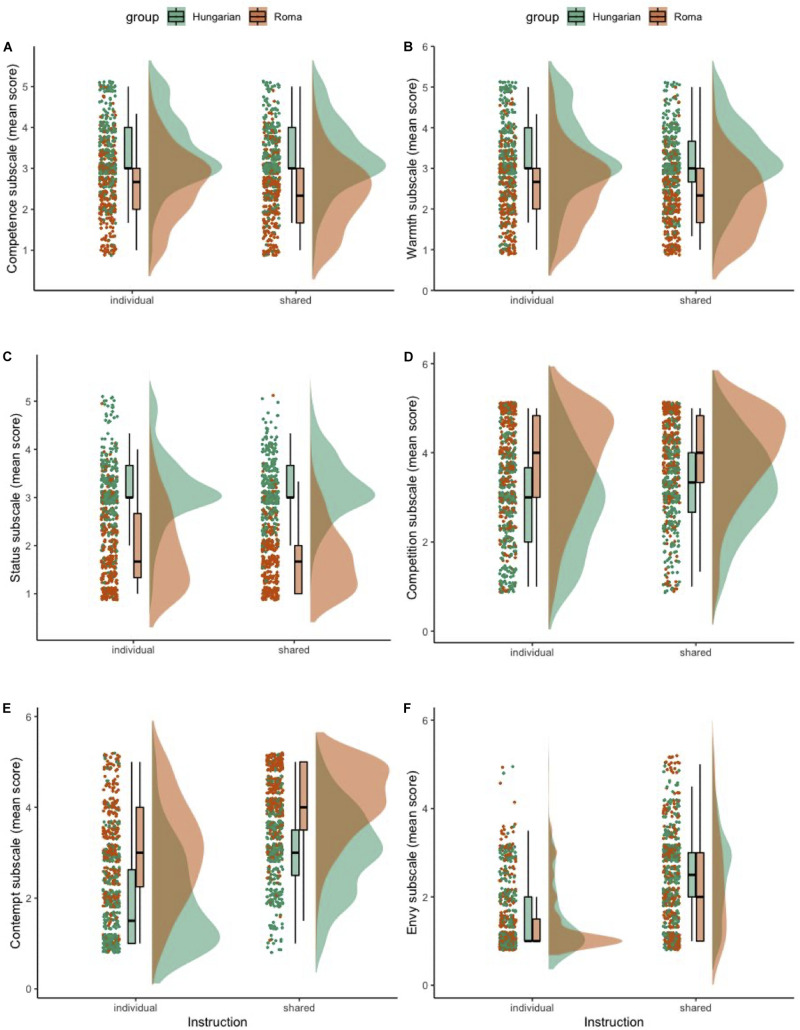
Individual responses for the BIAS map subscales per experimental factors, boxplots and distributions: competence **(A)**, warmth **(B)**, status **(C)**, competition **(D)**, contempt **(E)**, envy **(F)**.

**FIGURE 2:**
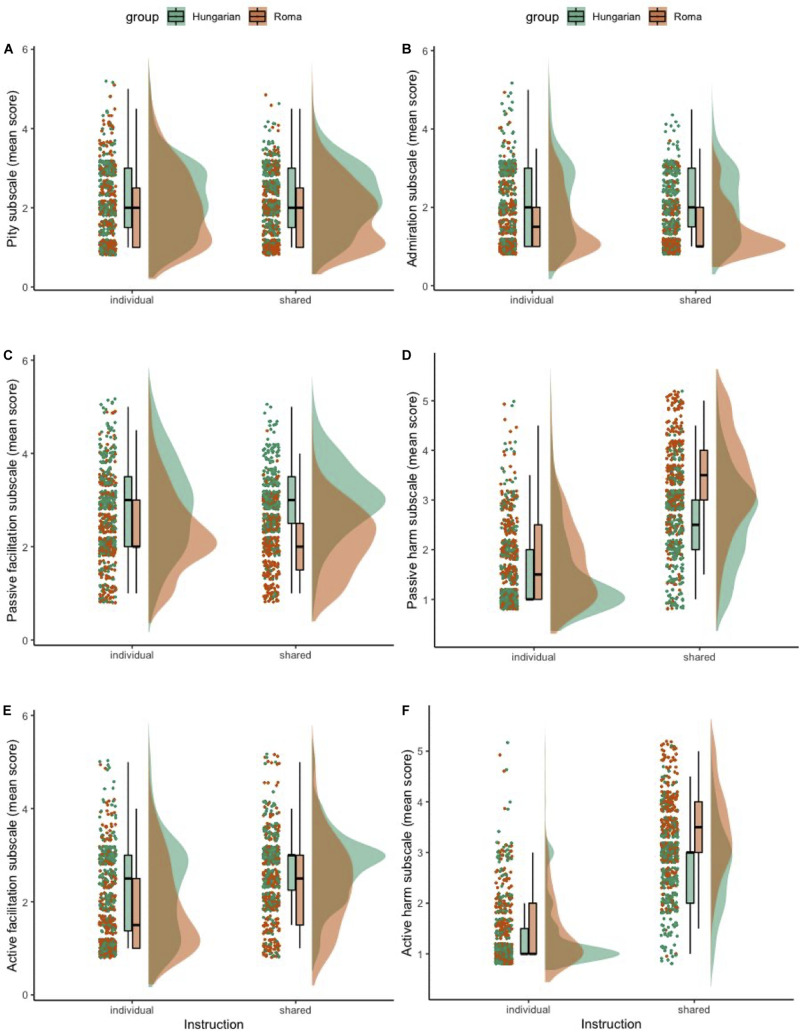
Individual responses for the BIAS map subscales per experimental factors, boxplots and distributions: pity **(A)**, admiration **(B)**, passive facilitation **(C)**, passive harm **(D)**, active facilitation **(E)**, active harm **(F)**.

In this section we firstly report the descriptive statistics of the BIAS map subscales, focusing on the differences between the scores obtained in the experimental groups. Secondly, we analyse the scalability and reliability properties of the BIAS map. Thirdly, we examine the measurement invariance of the BIAS map. Fourthly, we explore the relationship between the response instruction (individual vs. shared cultural perspective), target group (stigmatized vs. non-stigmatized out-group), and the mediation hypothesis underlying the BIAS map. Finally, we report the hypothesized impact of response instruction, target group, and their interaction on the BIAS map scales. Outcome variables can be visually inspected in Figures 1, 2 with respective boxplots and distributions.

### Descriptives

Following recommendations by Ho and Yu (2015) and Cain et al. (2017), we focus on the multivariate skewness and kurtosis of the BIAS map scales (see Tables 1, 2). We report means, standard deviations, skewness and kurtosis of the respective subscales in Supplementary Material. Multivariate skewness and kurtosis follow the same logic as univariate, but compare the joint distribution of multiple variables against a multivariate normal distribution (Cain et al., 2017). For both multivariate skewness and kurtosis, a test statistic and *p*-value were computed. A *p*-value smaller than 0.05 indicates a non-normal distribution of the joint population. Out of all the BIAS map scales (social structure, stereotypes, emotions and behavioral tendencies), only the social structure scale produced non-significant results when multivariate skewness was analyzed. Formally, this indicates a lack of evidence for the distribution’s departure from normality (Cain et al., 2017, p. 1718), but only in two out of the four experimental groups. In one case, the experimental group of “Hungarian + shared cultural perspective,” the finding overlaps with a non-significant result of the multivariate kurtosis test, suggesting a multivariate normal distribution. No other combination of scale and experimental condition produced non-significant results for multivariate skewness and for kurtosis. These results suggest that statistical tests that rely on normality assumptions could be negatively influenced by the underlying data. Descriptive statistics and visualizations, including distributions, means, SD and correlations are reported in Supplementary Material.

**TABLE 1:** Multivariate skewness of the BIAS map scales.

**Skew**	**Roma + shared cultural**			**Hungarian + shared cultural**			**Roma + individual**			**Hungarian + individual**		
	***b***	**z**	***p***	***b***	**z**	***p***	***b***	**z**	***p***	***b***	**z**	***p***
Social structure	2.10	111.54	<0.001	0.15	7.98	0.092	1.10	59.01	<0.001	0.09	4.89	0.299
Stereotypes	0.42	22.42	<0.001	0.31	16.23	0.003	0.35	18.73	0.001	0.29	15.11	0.004
Emotions	4.61	245.04	<0.001	1.07	55.23	<0.001	6.30	339.03	<0.001	4.68	246.43	<0.001
Behavioral tendencies	1.63	86.58	<0.001	1.26	65.11	<0.001	5.59	300.92	<0.001	9.11	479.57	<0.001

**TABLE 2:** Multivariate kurtosis of the BIAS map scales.

**Kurtosis**	**Roma + shared cultural**			**Hungarian + shared cultural**			**Roma + individual**			**Hungarian + individual**		
	***b***	**z**	***p***	***b***	**z**	***p***	***b***	**z**	***p***	***b***	**z**	***P***
Social structure	10.08	4.63	<0.001	8.25	0.55	0.581	7.45	–1.24	0.217	9.25	2.77	0.006
Stereotypes	8.63	1.41	0.158	10.04	4.50	<0.001	8.33	0.75	0.455	9.97	4.37	<0.001
Emotions	29.06	6.52	<0.001	25.71	2.18	0.029	31.09	9.20	<0.001	27.27	4.20	<0.001
Behavioral tendencies	27.74	4.82	<0.001	29.69	7.24	<0.001	32.27	10.73	<0.001	35.33	14.54	<0.001

### Reliability and Scalability

#### Stereotypes

Automated item selection procedure (AISP) from the *mokken* package (Andries van der Ar, 2012) showed that the perceived competence and warmth items fit into the respective subscales in all four experimental conditions (see Table 3 for details about the scalability of all scales). The *H* coefficients did not indicate any systemic problems with the scalability of the subscales, neither did the results of the reliability analysis using Cronbach’s *α* and McDonald’s *ω* coefficients (see Supplementary Tables 3–5). In all four experimental conditions was reliability of stereotypes subscales above 0.8 for Cronbach’s *α*, with the lowest score in the “Roma + shared cultural perspective” condition for competence subscale (Cronbach’s *α* = 0.8), not indicating any issues with the measures.

**TABLE 3:** Mokken *H* coefficients for respective experimental conditions and the BIAS map subscales.

**Subscale**	**Roma + shared cultural**		**Hungarian + shared cultural**		**Roma + individual**		**Hungarian + individual**	
	***H***	***SE H***	***H***	***SE H***	***H***	***SE H***	***H***	***SE H***
Competence	0.62	0.04	0.71	0.03	0.69	0.03	0.79	0.79
Warmth	0.66	0.04	0.71	0.03	0.72	0.04	0.79	0.02
Status	0.41	0.05	0.5	0.04	0.52	0.04	0.68	0.04
Competition	0.49	0.05	0.59	0.04	0.62	0.04	0.65	0.03
Contempt	0.35	0.07	0.6	0.05	0.56	0.05	0.71	0.05
Admiration	0.41	0.09	0.57	0.06	0.66	0.05	0.70	0.04
Pity	0.57	0.06	0.58	0.06	0.66	0.05	0.52	0.06
Envy	0.54	0.06	0.66	0.04	0.56	0.07	0.75	0.05
Active harm	0.14	0.07	0.41	0.06	0.41	0.08	0.67	0.07
Passive harm	0.44	0.06	0.56	0.05	0.47	0.07	0.75	0.05
Active facilitation	0.55	0.06	0.51	0.07	0.66	0.05	0.69	0.05
Passive facilitation	0.47	0.07	0.51	0.07	0.56	0.06	0.59	0.05

#### Social Structure

The Mokken scale analysis of the two subscales (status, competitiveness) using the AISP algorithm showed that the items form a joint scale in all four experimental conditions. Scalability was below 0.5 in both social structure subscales in one experimental condition (“Roma + shared cultural perspective”), indicating moderate scalability. Cronbach’s *α* and McDonald’s *ω* coefficients were acceptable in all conditions and subscales. Reliability of social structure subscales ranged from 0.64 and 0.71 (Cronbach’s *α*) in the “Roma + shared cultural perspective” condition to 0.83 and 0.84 (Cronbach’s *α*) in the “Hungarian + individual perspective” condition.

#### Emotions

Automated item selection procedure of emotions subscales showed that they can form individual scales; however, there was variation in scalability between the experimental conditions. Scalability ranged from 0.35 (weak scalability) for contempt in the “Roma + shared cultural perspective” condition to 0.75 (strong scalability) for envy in the “Hungarian + individual perspective” condition. A similar pattern was present in the other emotions subscales, with the exception of pity, which showed comparable scalability across conditions. Regarding the reliability of emotions subscales, subscales in the “Roma + shared cultural perspective” condition showed the lowest reliability (ranging from 0.46 to 0.7 Cronbach’s *α*), while data from the “Hungarian + individual perspective” condition produced the highest reliability (ranging from 0.59 to 0.81 Cronbach’s *α*).

#### Behavioral Tendencies

The Mokken scale analysis showed that active harm was not scalable in the “Roma + shared cultural perspective” condition (H = 0.14). In the other experimental conditions, active harm showed relatively low scalability, compared to the other behavioral tendencies subscales. Active harm had the lowest reliability of the measures, ranging from a mere Cronbach’s *α* = 0.22 for active harm subscale in the “Roma + shared cultural perspective” condition to acceptable levels above the 0.6 threshold for all 4 subscales in the “Hungarian + individual perspective” condition.

#### Invariance of the BIAS Map

To analyze measurement invariance, we used lavaan (Rosseel, 2012; Cheung, 2015) and semTools (Jorgensen et al., 2020) packages. Due to having an empty category in one of the variables (no participant had chosen the point 4 on a 5-point scale in the “Hungarian + individual perspective” condition for an “envy” questionnaire item), we were not able to use analysis suitable for categorical data, but resorted to using a MLR estimator to obtain robust standard errors and test statistics. CFA model included all 12 BIAS map subscales, defined as latent variables. The model indices [χ^2^(1,136) = 2010.508, *p* < 0.001, robust RMSEA = 0.051, 90% CI [0.048, 0.055], robust CFI = 0.937, robust TLI = 0.916] suggest a mixed evidence regarding goodness of its fit. The invariance test supported metric invariance of the model, but not scalar, nor mean invariance. Fits of all models are reported in Table 4. These results suggest that participants saw the same meaning in the latent constructs across experimental conditions, but absence of full equivalence prevents from directly comparing means without further considerations (Fischer and Karl, 2019).

**TABLE 4:** Model indices for measurement invariance across experimental conditions.

**Model**	***df***	**χ^2^**	***p***	**CFI scaled**	**RMSEA scaled**
Configural	1,136	2,174		0.932	0.049
Metric	1,184	2,272	0.19	0.931	0.049
Scalar	1,232	2,931	<0.001	0.887	0.061
Mean	1,268	4,657	<0.001	0.767	0.086

### Differences in the BIAS Map Scales

We visually observed differences in the content of most of the BIAS map scales for participants in both the individual and shared cultural perspectives, as well as in both target group conditions (Roma and Hungarian) (see Figures 1, 2). A *MANOVA.wide* function was used to calculate Wald-type statistics (WTS) and resampled test statistics (1000 iterations for calculating resampled statistics). See Tables 5–8 for statistical details. For interpretation of statistical tests (interactions and main effects), we used a corrected α level of 0.0048.

**TABLE 5:** Results of the non-parametric multivariate MANOVA-like test, including *post hoc* pairwise comparisons, for social structure subscales (status, competitiveness).

**Predictors**	**WTS test statistic**	***df***	***p***	**Resampled *p***
Instruction	22.193	2	< 0.001	<0.001
Group	1286.909	2	< 0.001	<0.001
Instruction:group	13.211	2	0.001	0.001

**Factor pairwise comparison**	**Contrast *p***	**Estimate**	**CI lower**	**CI upper**

Individual Roma − shared Hungarian	<0.001	−0.712	−1.106	−0.318
Individual Roma − shared Roma	0.994	0.036	−0.362	0.434
Individual Roma − individual Hungarian	0.374	−0.265	−0.683	0.153
Individual Hungarian − shared Hungarian	0.017	−0.447	−0.838	−0.056
Individual Hungarian − shared Roma	0.208	0.301	−0.094	0.696
Shared Roma − shared Hungarian	< 0.001	−0.748	−1.117	−0.379

**TABLE 6:** Results of the non-parametric multivariate MANOVA-like test, including *post hoc* pairwise comparisons, for stereotypes subscales (competence, warmth).

**Predictors**	**WTS test statistic**	***df***	***p***	**Resampled *p***
Instruction	17.665	2	<0.001	<0.001
Group	373.992	2	<0.001	<0.001
Instruction:group	4.215	2	0.122	0.124

**Factor pairwise comparison**	**contrast *p***	**Estimate**	**CI lower**	**CI upper**

Individual Roma − individual Hungarian	<0.001	−1.543	−2.055	−1.031
Individual Roma − shared Roma	0.016	0.557	0.069	1.045
Individual Roma − shared Hungarian	<0.001	−1.348	−1.836	−0.860
Individual Hungarian − shared Hungarian	0.770	0.195	−0.309	0.699
Individual Hungarian − shared Roma	<0.001	2.100	1.596	2.604
Shared Roma − shared Hungarian	<0.001	−1.905	−2.385	−1.425

**TABLE 7:** Results of the non-parametric multivariate MANOVA-like test, including *post hoc* pairwise comparisons, for emotion subscales (contempt, admiration, pity, envy).

**Predictors**	**WTS test statistic**	***df***	***p***	**Resampled *p***
Instruction	621.219	4	<0.001	<0.001
Group	506.249	4	<0.001	<0.001
Instruction:group	8.772	4	0.067	0.072

**Factor pairwise comparison**	**Contrast *p***	**Estimate**	**CI lower**	**CI upper**

Individual Roma − individual Hungarian	0.307	0.416	−0.208	1.040
Individual Roma − shared Roma	<0.001	−1.538	−2.140	−0.936
Individual Roma − shared Hungarian	<0.001	−1.693	−2.306	−1.080
Individual Hungarian − shared Hungarian	<0.001	−2.109	−2.729	−1.490
Individual Hungarian − shared Roma	<0.001	−1.954	−2.563	−1.345
Shared Roma − shared Hungarian	0.910	−0.155	−0.753	0.443

**TABLE 8:** Results of the non-parametric multivariate MANOVA-like test, including *post hoc* pairwise comparisons, for behavioral tendencies subscales (active and passive harm, active and passive facilitation).

**Predictors**	**WTS test statistic**	***df***	***p***	**Resampled *p***
Instruction	1754.081	4	<0.001	<0.001
Group	310.037	4	<0.001	<0.001
Instruction:group	72.865	4	<0.001	<0.001

**Factor pairwise comparison**	**Contrast *p***	**Estimate**	**CI lower**	**CI upper**

Individual Roma − individual Hungarian	0.030	−0.620	−1.197	−0.043
Individual Roma − shared Hungarian	<0.001	−3.649	−4.202	−3.096
Individual Roma − shared Roma	<0.001	−3.928	−4.494	−3.362
Individual Hungarian − shared Hungarian	<0.001	−3.029	−3.606	−2.452
Individual Hungarian − shared Roma	<0.001	−3.308	−3.897	−2.719
Shared Roma − shared Hungarian	0.588	0.279	−0.287	0.845

#### Social Structure

A two-way multivariate analysis was conducted that examined the effect of instruction and target group on social structure subscales (status, competition; see Figures 1C,D). There was a statistically significant interaction between the effects of the target group and instruction, WTS(*df* = 2) = 13.21, *p* = 0.001. Main effects analysis showed an effect of both the instruction [WTS(*df* = 2) = 22.19, *p* < 0.001] and target group [WTS(*df* = 2) = 1286.9, *p* < 0.001]. Multivariate *post hoc* comparisons using Tukey’s all-pairwise comparisons showed statistically significant differences between “Roma + shared cultural perspective” and “Hungarian + shared cultural perspective” (*p* < 0.001, summary effect estimate averaged over all dimensions = −0.75) combination of factors; “Hungarian + individual perspective” and “Hungarian + shared cultural perspective” (*p* = 0.017, effect estimate = −0.45); and between “Roma + individual perspective” and “Hungarian + shared cultural perspective” (*p* < 0.001, effect estimate = −0.71) combinations of factors.

#### Stereotypes

Examining the effect of experimental factors on stereotypes subscales (competence, warmth; see Figures 1A,B), there was a statistically non-significant interaction between the effects of the target group and instruction, WTS(*df* = 2) = 4.215, *p* = 0.122. Main effects analysis showed an effect of both the instruction [WTS(*df* = 2) = 17.66, *p* < 0.001] and target group [WTS(*df* = 2) = 374, *p* < 0.001].

#### Emotions

There was also a non-significant interaction between the effect of instruction and target group on emotions subscales (contempt, admiration, pity, envy; see Figures 1E,F, 2A,B), WTS(*df* = 4) = 8.77, *p* = 0.067. Main effects analysis showed an effect of both the instruction [WTS(*df* = 4) = 621.22, *p* < 0.001] and target group [WTS(*df* = 4) = 506.25, *p* < 0.001].

#### Behavioral Tendencies

There was a statistically significant interaction between the effect of instruction and target group on behavioral tendencies subscales (active and passive facilitation, active and passive harm; see Figures 2C–F), WTS(*df* = 4) = 72.87, *p* < 0.001. Main effects analysis showed an effect of both the instruction [WTS(*df* = 4) = 1754.08, *p* < 0.001] and target group [WTS(*df* = 4) = 310.04, *p* ≤ 0.001]. Multivariate *post hoc* comparisons using Tukey’s all-pairwise comparisons showed statistically significant differences between “Hungarian + individual perspective” and “Hungarian + shared cultural perspective” combination of factors (*p* < 0.001; summary effect estimate averaged over all dimensions = −3.03); “Roma + individual perspective” and “Hungarian + shared cultural perspective” (*p* < 0.001, effect estimate = −3.65); “Hungarian + individual perspective” and “Roma + shared cultural perspective” (*p* < 0.001, effect estimate = −3.31); “Roma + individual perspective” − “Roma + shared cultural perspective” (*p* < 0.001, effect estimate = −3.93); and between “Roma + individual perspective” and “Hungarian + individual perspective” combination of factors (*p* = 0.037, −0.62).

### Mediation Analysis

For each experimental condition, we computed four parallel multiple mediator models separately using the *mediate* function from psych package (Revelle, 2019). To evaluate the presence or absence of a mediating relationship, we used bootstrapped (10,000 samples) indirect effects (total effects, direct effects as well as bootstrapped indirect effects are reported in Supplementary Tables 4–7). In this analysis, we used a parametric approach, built on linear regression, initially proposed to evaluate mediation hypotheses in the BIAS map model (Cuddy et al., 2007). Cuddy et al. (2007), Studies 2 and 3 presented experimental evidence supporting a causal relationship between stereotypes and emotions, and stereotypes and behavioral tendencies. In line with previous replications (Bye and Herrebrøden, 2018), adopting this approach allows us to compare our analysis with previously published results. Following the advice of Fiedler et al. (2018) we acknowledge that the significant results of the mediation in the present study are conditional on the BIAS map model’s hypothesis of a causal relationship between stereotypes, emotions, and behavioral tendencies. Likewise, we acknowledge that other models of their relationship cannot be excluded.

With the exception of the “Roma + shared cultural perspective” experimental condition, a higher perceived warmth was associated with less active harm as a result of the effect of warmth on contempt, which in turn influenced levels of active harm (bootstrapped indirect effect of warmth ranged from *b* = −0.2 to −0.05; bootstrapped indirect effect via contempt ranged from *b* = −0.2 to −0.05). There was no evidence that feelings of envy mediated the negative association between warmth and active harm.

In all four experimental conditions, a higher perceived warmth was associated with higher active facilitation as a result of the effect of warmth on admiration and pity, which in turn influenced behavioral tendencies (bootstrapped indirect effect of warmth ranged from *b* = 0.09 to 0.3; bootstrapped indirect effect via admiration ranged from *b* = 0.07 to 0.15; bootstrapped indirect effect via pity ranged from *b* = 0.02 to 0.15). The mediating mechanism in the “Roma + shared cultural perspective” condition was present only for pity, but there was no evidence of the mediating mechanism for admiration.

With the exception of the “Roma + shared cultural perspective” experimental group, higher perceived competence was associated with less passive harm as a result of the effect of competence on contempt, which in turn influenced levels of passive harm (bootstrapped indirect effect of competence ranged from *b* = −0.08 to −0.25; bootstrapped indirect effect via contempt ranged from *b* = −0.02 to −0.26; bootstrapped indirect effect via pity ranged from *b* = −0.06 to −0.26). The effect of competence on passive harm was mediated through feelings of pity in the “Roma + shared cultural perspective” condition.

In all four experimental conditions, higher perceived competence was associated with less passive facilitation as a result of the effect of competence on admiration, which in turn influenced levels of passive facilitation (bootstrapped indirect effect of competence ranged from *b* = 0.06 to 0.15; bootstrapped indirect effect via admiration ranged from *b* = 0.06 to 0.15). There was no evidence that feelings of envy mediated the association between competence and passive facilitation.

## Discussion

The results support H1 and H2, and partially support H3. They show that response instruction (H1) and target group (H2) had significant effects on scores in the BIAS map scales. Furthermore, they reveal a significant effect of interaction (H3) between the response instruction and target group on scores in social structure and behavioral tendencies (but not stereotypes and emotions) BIAS map scales. The results also suggest partial influence on the mediation hypothesis underlying the BIAS map; and minor influence on its scale properties.

### The Impact of Response Instruction and Target Group on Scale Properties

There were only small differences between the experimental conditions in the *scale properties* of the BIAS map subscales, with the notable exception of the “Roma + shared cultural perspective” condition, which displayed the lowest levels of scalability and reliability. Its social structure subscales (status, competitiveness) and two behavioral tendencies subscales (passive harm, passive facilitation) had moderate scalability, two emotions subscales (contempt, admiration) had low scalability, and one behavioral tendencies subscale (active harm) was not scalable. In all the experimental groups, active harm was the least scalable and reliable subscale of the BIAS map.

The least satisfactory scale properties in the “Roma + shared cultural perspective” experimental condition can be partly explained by participants’ perceptions of the contradictory social norms associated with the Roma in Slovakia, whose polarizing effect could have rendered a normal data distribution impossible. These perceptions could reflect the contrast between the normative approval of anti-Roma stereotypes, prejudice and discrimination, most visible in the infra-humanizing language to which the Roma are subjected in political discourse (Kluknavská, 2013; Kroon et al., 2016) and the human rights protection and anti-discrimination norms enshrined in domestic and especially European Union legislation (Chopin et al., 2017).

Similarly, the fact that active harm was the least scalable and reliable subscale of the BIAS map could be related to the ambiguous normative perceptions of the Roma as a category of people who suffer from both verbal and physical conflicts with ethnic Slovaks. Although the Roma are often the victims of police violence (Szilvasi et al., 2013; The Slovak Spectator, 2017), they are also frequently represented as inherently vicious, immoral and inclined to criminal behavior (Tileagă, 2006; Kroon et al., 2016).

Scalability issues of some BIAS map subscales (e.g., active harm) could indicate problems with ecological validity. The problematic items need to be cross-culturally validated using both quantitative and qualitative (e.g., cognitive interviews) methods to identify reasons for their unsatisfactory performance and suggest potential modifications (Lášticová et al., underv review). The validation process could lead to development of a more target-group tailored measure of stereotypes that would capture the specific position of the target group within the culture-specific context of intergroup relations (Bu and Borgida, 2020). A mixed-methods approach could also be helpful in exploring how and why contradictory social norms might affect some but not all dimensions of the BIAS map, and why some dimensions of the BIAS map are more and other less susceptible to normative influence.

### The Influence of Response Instruction and Target Group on Mediation Hypothesis

Our findings partly challenge the *mediation hypothesis* proposed for the BIAS map measure (Cuddy et al., 2007). In three out of the four behavioral tendencies subscales, the behavioral tendencies were in most sub-groups mediated by a single emotion, passive facilitation being the sole exception. This is mostly in line with Bye and Herrebrøden (2018) and Constantin and Cuadrado (2019), who report that “for each of the four behavior outcomes the effect of stereotype content was mediated through one emotion rather than two as predicted by the BIAS map” (p. 1). The mediation models proposed for the BIAS map measure performed furthest from theoretical predictions in the “Roma + shared cultural perspective” experimental condition. In contrast to other experimental conditions, there was no evidence of the mediating mechanism in two out of four behavioral tendencies subscales (active harm and passive harm) in the “Roma + shared cultural perspective” experimental condition. The difference could be attributed to the effects of the response instruction and the target group as well as to the limited reliability and scalability of the BIAS map in the “Roma + shared cultural perspective” experimental condition. However, since we were not directly testing differences between mediation models in respective experimental conditions, our findings must be viewed with caution and should be further investigated with a new data collection in a future research.

### The Impact of Response Instruction on the BIAS Map Scores

The systematic differences between participants’ responses when instructed to give answers from either their own individual perspective or the shared cultural perspective for both target groups point to *the effect of the response instruction* on the BIAS map measure (H1). The more pervasive difference between individual stereotypical beliefs and perceptions of shared cultural stereotypes in relation to the Roma rather than the Hungarians could indicate differences in the perceived social consensus (Haslam et al., 1996; Stangor et al., 2001b). They could suggest that there is actually a normative dissensus—relative to their personal opinions, participants perceive social norms relating to the Roma as more ambivalent and perhaps contradictory than those relating to the Hungarians. In the present study, the unsatisfactory scale properties of the “Roma + shared cultural perspective” experimental condition, which violate the assumptions of normality, give support to the latter interpretation. These findings extend those of Kotzur et al. (2020) to all dimensions of the BIAS map model. Based on their findings, Kotzur et al. (2020) proposed “aggregating stereotype content scores from participants’ personal perspective to the cultural level.” In contrast, we argue that instructing participants to respond from a shared cultural perspective can reveal the social normative consensus or dissensus in the social perception of the target group (e.g., the Roma) that responding from an individual perspective is unable to provide. On the other hand, when seeking to measure individuals’ stereotypical beliefs about target groups (e.g., when testing the effectiveness of prejudice reduction interventions), instruction from a personal perspective seems to be an adequate choice. In fact, a comparison between individual stereotypical beliefs and perceptions of shared cultural stereotypes could become a useful operationalization for assessing the “normative climate” (Váradi, 2014; Forscher et al., 2015) or “normative context” (Kende et al., 2017; Kende and McGarty, 2019). The concept of “normative climate” would allow for studying the attitude-social norm context in which stereotypes and prejudice toward different target groups are expressed or withheld.

### The Impact of Target Group on the BIAS Map Scores

The observed *effect of the target group* on the scores of the BIAS map measure (H2) is an expected finding because the BIAS map and the SCM were developed to measure the content of stereotypical beliefs and related social structure, emotions and behavioral tendencies toward various target groups. This is in line with the findings of Kende et al. (2020) who report blatant negative stereotyping of the Roma across six European countries. In Hungary, Romania, and Slovakia, the Roma were also perceived as competitors for limited resources, receiving undeserved benefits (Kende et al., 2020). Participants also expressed stronger tendencies to exclude and demean them (higher in passive harm); a weaker inclination to cooperate with and associate with them (lower in passive facilitation); and to help and protect them (lower in active facilitation) than they did in relation to the Hungarians. These findings provide additional supporting evidence to previous literature characterizing the Roma as a low status, stigmatized, dehumanized out-group, subjected to the expression of blatant prejudice and discrimination (Kteily et al., 2015; Kende et al., 2017, Study 4) and low collective action intentions concerning the Roma in Slovakia (Poslon et al., 2020).

More notably, these findings also underscore the observed effect of the response instruction on the BIAS map—the effect of the target group on emotions and behavioral tendencies was more evident when participants were instructed to respond to questions from the shared cultural perspective than from the individual perspective.

### Interaction of the Impact of Response Instruction and Target Group on the BIAS Map Scores

The results partially support hypothesis about *interaction effect of response instruction and target group* on the BIAS maps scores (H3): stigmatized target group (Roma) elicited less favorable evaluations in social structure and behavioral tendencies (but not in stereotypes and emotions) scales when reported from a shared cultural perspective (compared to individual perspective) than non-stigmatized target group (Hungarian). There was no interaction effect of response instruction and target group on stereotypes and emotions scales of the BIAS map. Responses from shared cultural perspective yielded less favorable stereotypical and affective evaluations than responses given from individual perspective irrespective of target groups being studied. However, there was a combined effect of these two factors on social structure and behavioral tendencies scales. Examination of pairwise comparisons suggests different patterns of interaction effects for each of these factors.

In the case of social structure subscales (status, competitiveness) there were statistically significant differences between “Roma + shared cultural perspective” and “Hungarian + shared cultural perspective” conditions but not between “Roma + individual perspective” and “Roma + shared cultural perspective” and between “Roma + individual perspective” and “Hungarian + individual perspective” conditions. These findings give further credence to the role of divergent normative climates for Roma and for Hungarians in shaping the shared cultural perceptions of both target groups’ status and competitiveness.

In contrast, behavioral tendencies subscales (active and passive harm, active and passive facilitation) demonstrate a reversed pattern: there were statistically significant differences between all other conditions but not between “Roma + shared cultural perspective” and “Hungarian + shared cultural perspective” conditions. These findings suggest that inclinations to behave toward the members of Roma and Hungarian are less subject to shared normative concern and are more prone to individual beliefs.

However, we advise caution when interpreting these findings since they are limited to two target groups in Slovakia. Future research could attempt to replicate these findings with a larger number of different ethnic target groups in Slovakia (Ruthenians, Czechs, Ukrainians) or with target groups in different intergroup contexts.

### Treating Personal and Shared Cultural Stereotypes as Two Potentially Separate Constructs

In sum, the effects of response instruction and target group suggest that use of the individual perspective, as opposed to the shared cultural perspective response instruction, solicits different responses to the BIAS map and the SCM, especially in relation to target groups for whom stereotyping and prejudice is more normatively approved. Based on these findings, we argue for caution when using the individual perspective response instruction to measure the perceived normative perspective of most of the people in a society. Depending on the target group in question, instructing the participants to respond from their own individual perspective instead of from the shared cultural perspective of their society can significantly distort the outcomes produced by the BIAS map and the SCM and seriously undermine their construct validity as measures of shared cultural stereotypes. Conversely, identical concerns apply to using a shared cultural perspective response instruction to assess participants’ personal stereotypical beliefs. Our evidence gives further credence to treating individual stereotypes and shared cultural stereotypes as two potentially separate constructs with unique characteristics. However, further research is needed to ascertain their relative independence, i.e., the extent to which they are separate or interdependent.

### Limitations

There are two major limitations to our study.

First, the findings are limited to the context of ethnic intergroup relations in Slovakia. They need to be validated in different national and intergroup contexts, in which the same target groups (Roma, Hungarian) are imbued with different normative concerns (Bilewicz, 2012). Moreover, future research including typologically different target groups (e.g., national, age, gender) that are exposed to varied normative climates in different countries could provide a more robust test of the impact of the response instruction on the BIAS map and its interaction with target group type. For example, the awareness about the prevalence of stereotypes about target groups is an important source of normative information (Tankard and Paluck, 2016). Especially, when evidence suggests that awareness about the prevalence of stereotypes condones stereotyping and stereotype-consistent behavior (Duguid and Thomas-Hunt, 2015). Examining the sources of normative information and their relationship to personal stereotyping could help to illuminate both within- and between-culture variation in expression of stereotypes, and ultimately reinvigorate the role of (normative) context in the study of intergroup relations (Pettigrew, 2018).

The second limitation is the problematic reliability and scalability of the BIAS map scales and subscales, especially those pertaining to the normatively ambiguous beliefs, emotions and behavioral tendencies toward the Roma target group. The uneven reliability and scalability of the BIAS map scales and subscales is related to the skewness and kurtosis of the data, suggesting variation in distribution. While the “Hungarian + shared cultural perspective” experimental condition had a multivariate normal distribution for a single BIAS map scale (social structure), the data in the other experimental conditions violated these normality assumptions. However, it is difficult to assert whether the heterogeneity in the normal distribution of the data applies specifically to the sample characteristics and target groups used in the present study or whether it has also been found in other previously published studies in general. To our knowledge, it is not common practice to report the skewness and kurtosis of the scales and subscales in BIAS map (and SCM) studies, despite these distribution characteristics helping determine whether the data should be analyzed using parametric or non-parametric statistical tests. This practice could also influence the results of published experimental studies—a statistically “not significant” result could be down to multivariate skewness and kurtosis and the use of inappropriate statistical tests. Use of more suitable procedures could lead to the opposite conclusion, flipping the result into “significant” territory. More systematic reporting of the normal or non-normal data distribution in BIAS map and SCM studies could lead to superior cross-cultural and cross-target group comparisons and provide a more rigorous framework for testing the universal applicability of the BIAS map and the SCM.

## Conclusion

In conclusion, the present study provides novel evidence about the partial effects of response instruction, target group, and their interaction on scores, scale properties and the mediation hypothesis underlying the BIAS map measure. Rather than viewing the individual perspective response instruction as a threat to accuracy and construct validity of the BIAS map and the SCM as the measures of culturally shared perceptions of social structure, stereotypes, emotions, and behavioral tendencies, we argue for treating individual stereotypes and shared cultural stereotypes as two potentially separate constructs.

## Data Availability Statement

The datasets presented in this study can be found in online repositories. The names of the repository/repositories and accession number(s) can be found below: https://osf.io/h39xy/.

## Ethics Statement

The studies involving human participants were reviewed and approved by the Ethics Committee of the Institute for Research in Social Communication of the Slovak Academy of Sciences. The patients/participants provided their written informed consent to participate in this study.

## Author Contributions

AF, BL, and MP contributed to the conception and design of the study. MH analyzed the data. All authors wrote and revised the manuscript, and read and approved the submitted version.

## Conflict of Interest

The authors declare that the research was conducted in the absence of any commercial or financial relationships that could be construed as a potential conflict of interest.

## Footnotes

1 We will report the findings about the quantity and quality of the direct contact with the Roma and motivation to express prejudice and respond without prejudice in different paper. We will follow the recommendations of Kirkman and Chen (2011) and Colquitt (2013) to avoid the “data slicing” concerns. All other measures, manipulations and exclusion are disclosed and reported. The methods section details how the final sample size was determined. No data were collected after the analysis.
