# Supplementary material for: The Impact of Response Instruction and Target Group on the BIAS Map
Source: Front Psychol. 2020 Oct 6;11:566725. doi: 10.3389/fpsyg.2020.566725 (PMC7573118; doi:10.3389/fpsyg.2020.566725)
Supplement: Supplementary file 1 [file Data_Sheet_1.pdf]

Supplementary Material

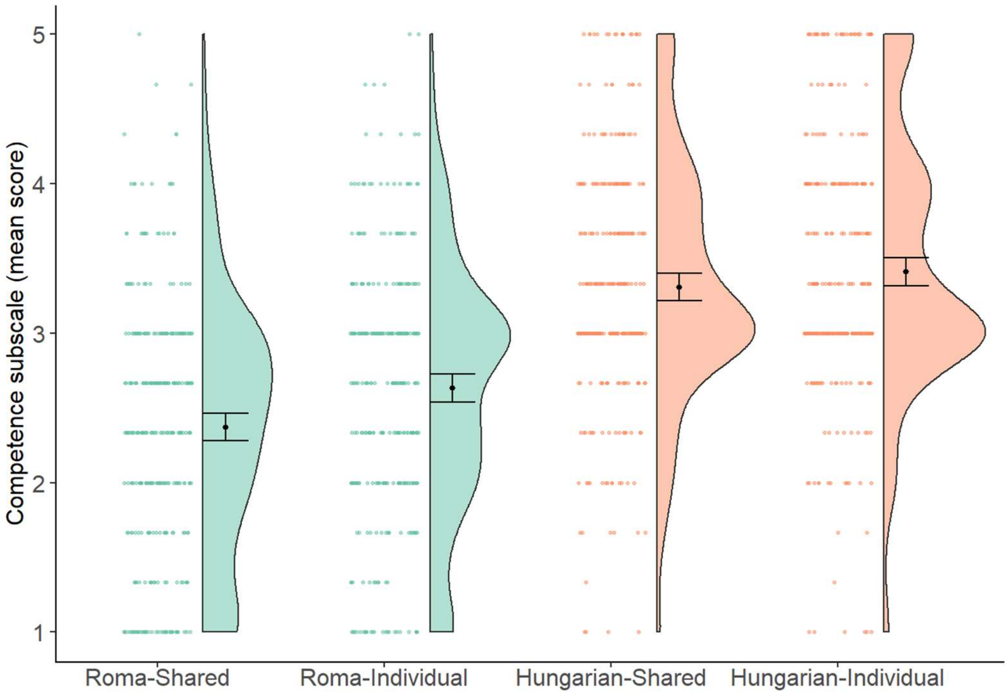

Supplementary Figure 1. Distributions, means and 95% confidence interval of **competence** subscale in respective experimental conditions.

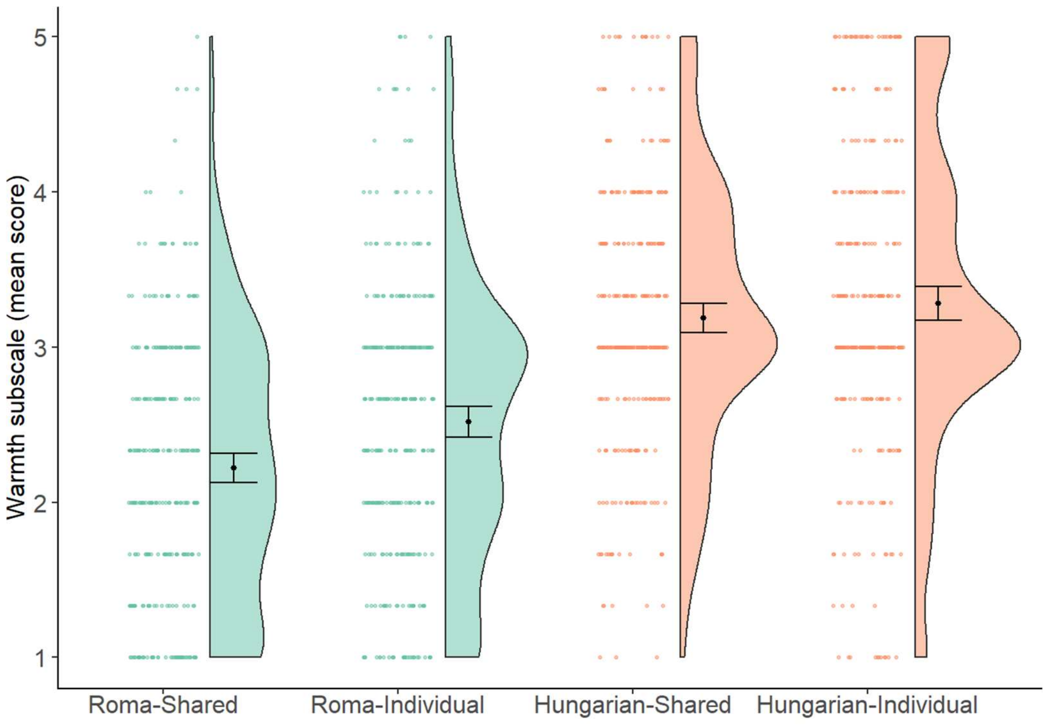

Supplementary Figure 2. Distributions, means and 95% confidence interval of **warmth** subscale in respective experimental conditions.

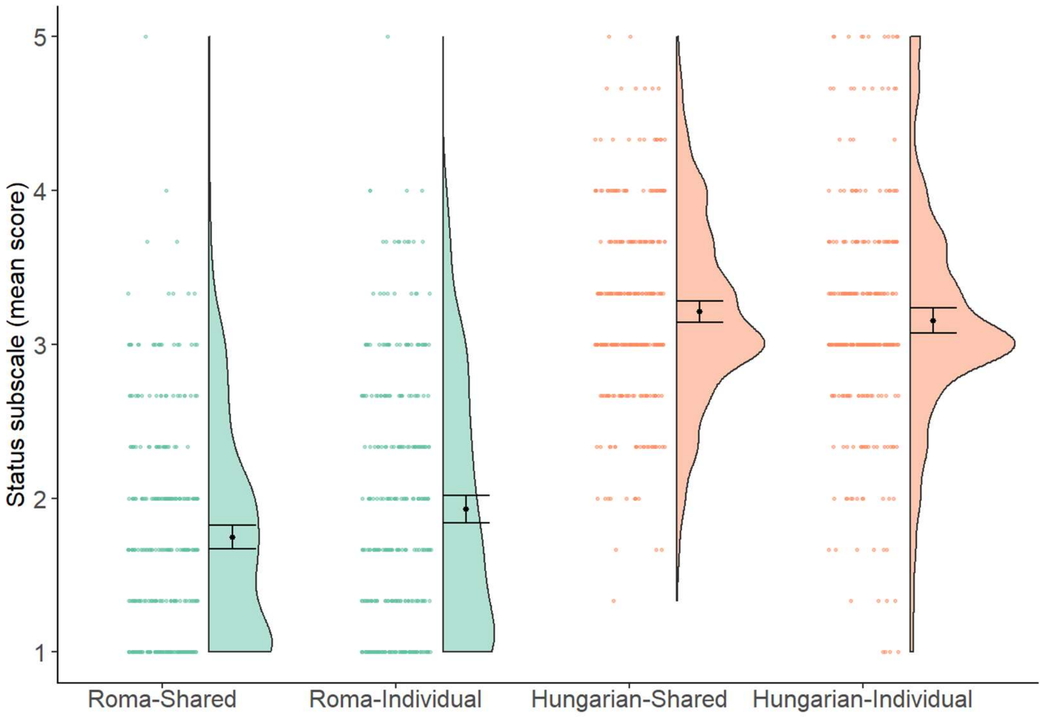

Supplementary Figure 3. Distributions, means and 95% confidence interval of **status** subscale in respective experimental conditions.

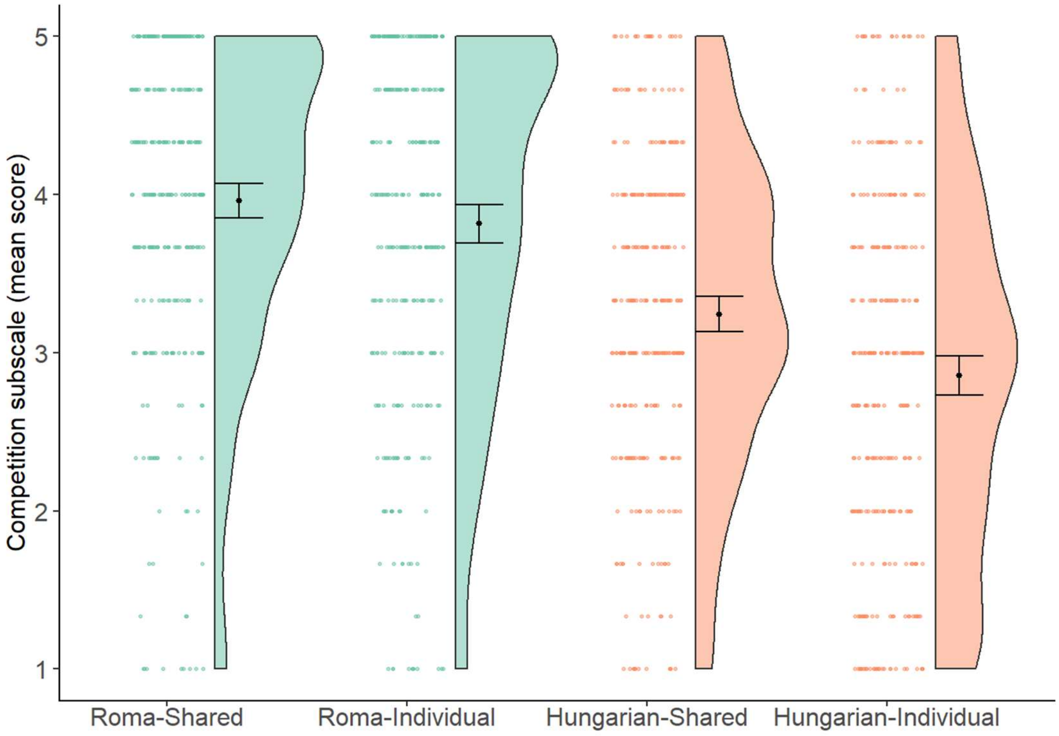

Supplementary Figure 4. Distributions, means and 95% confidence interval of **competition** subscale in respective experimental conditions.

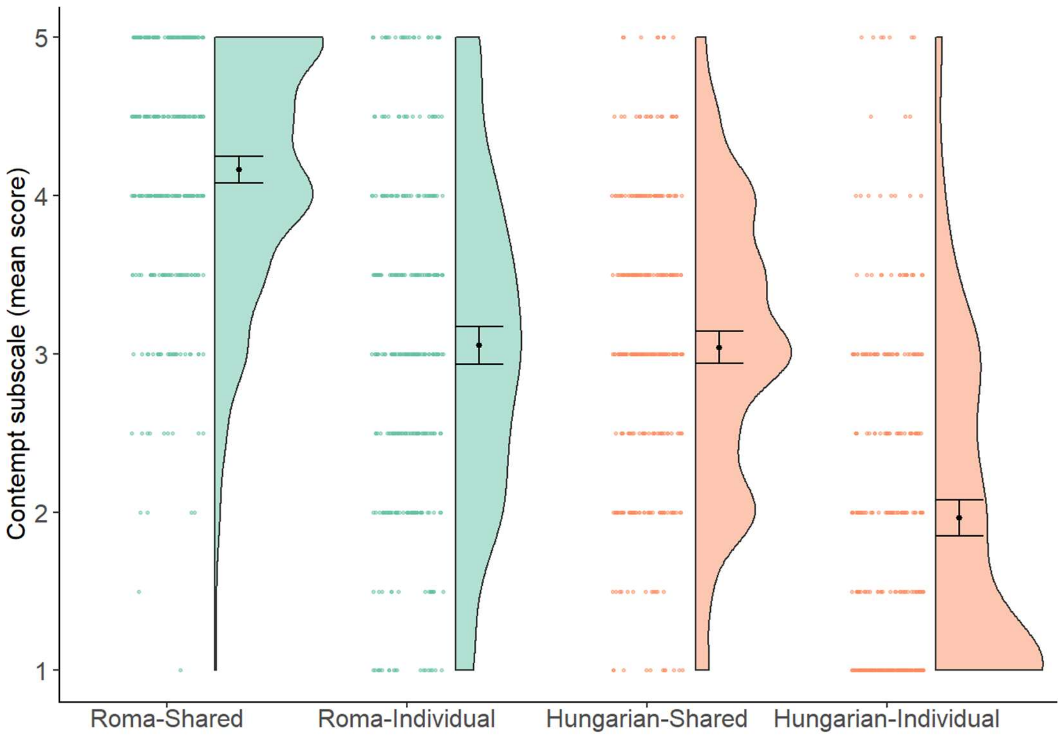

Supplementary Figure 5. Distributions, means and 95% confidence interval of **contempt** subscale in respective experimental conditions.

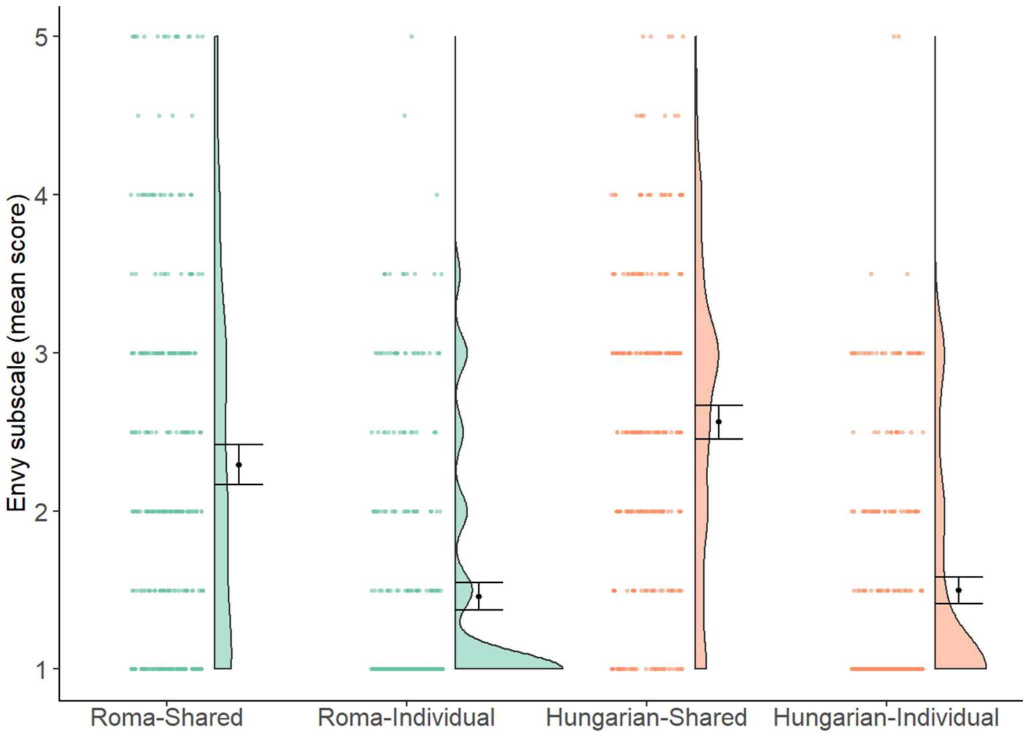

Supplementary Figure 6. Distributions, means and 95% confidence interval of **envy** subscale in respective experimental conditions.

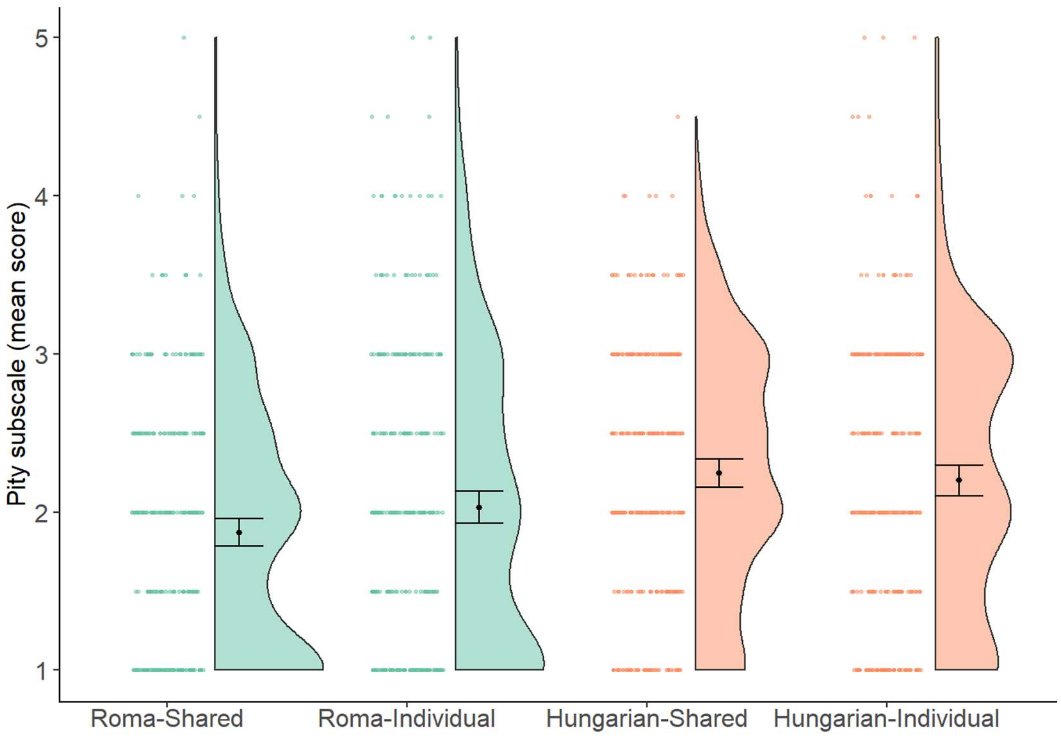

Supplementary Figure 7. Distributions, means and 95% confidence interval of **pity** subscale in respective experimental conditions.

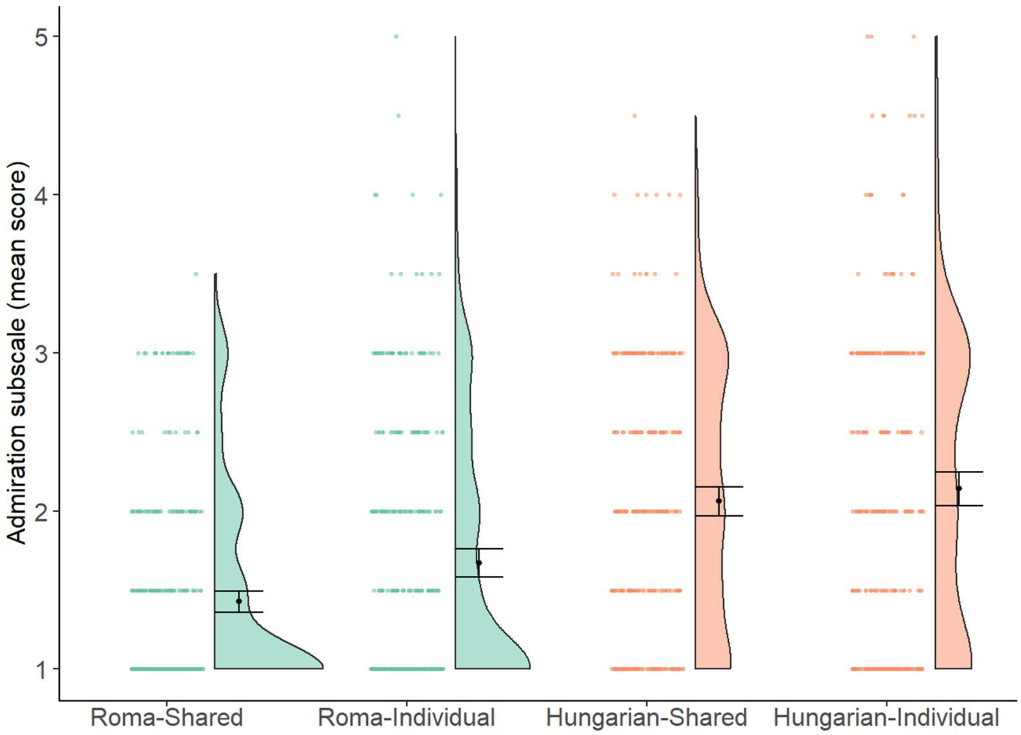

Supplementary Figure 8. Distributions, means and 95% confidence interval of **admiration** subscale in respective experimental conditions.

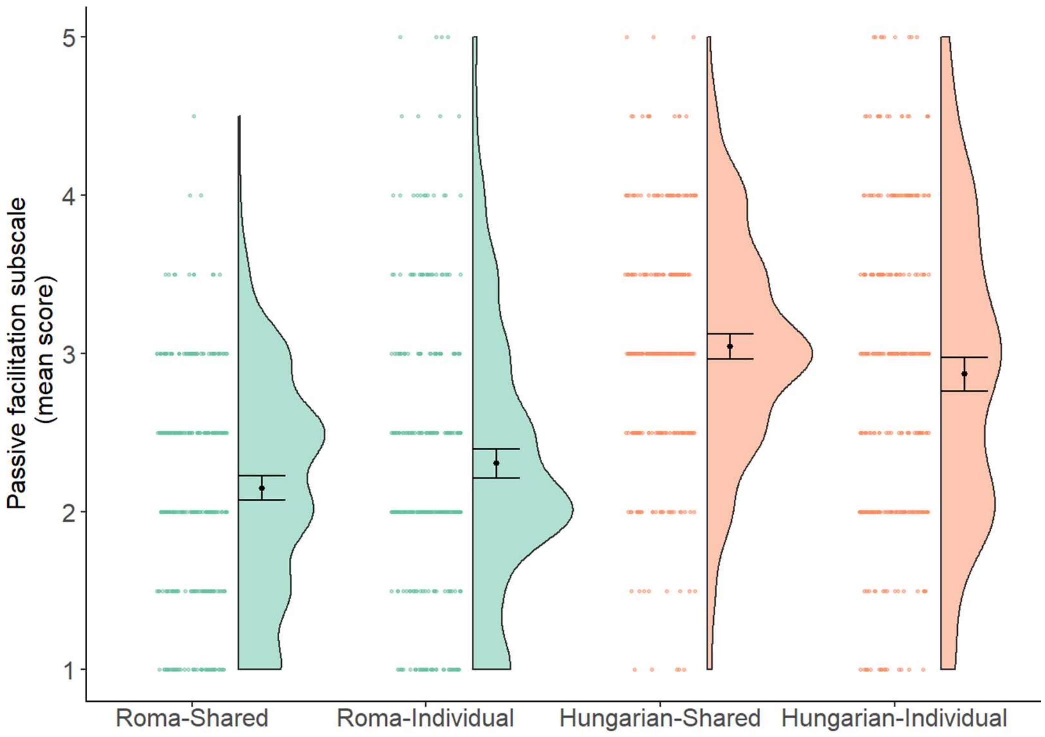

Supplementary Figure 9. Distributions, means and 95% confidence interval of **passive facilitation** subscale in respective experimental conditions.

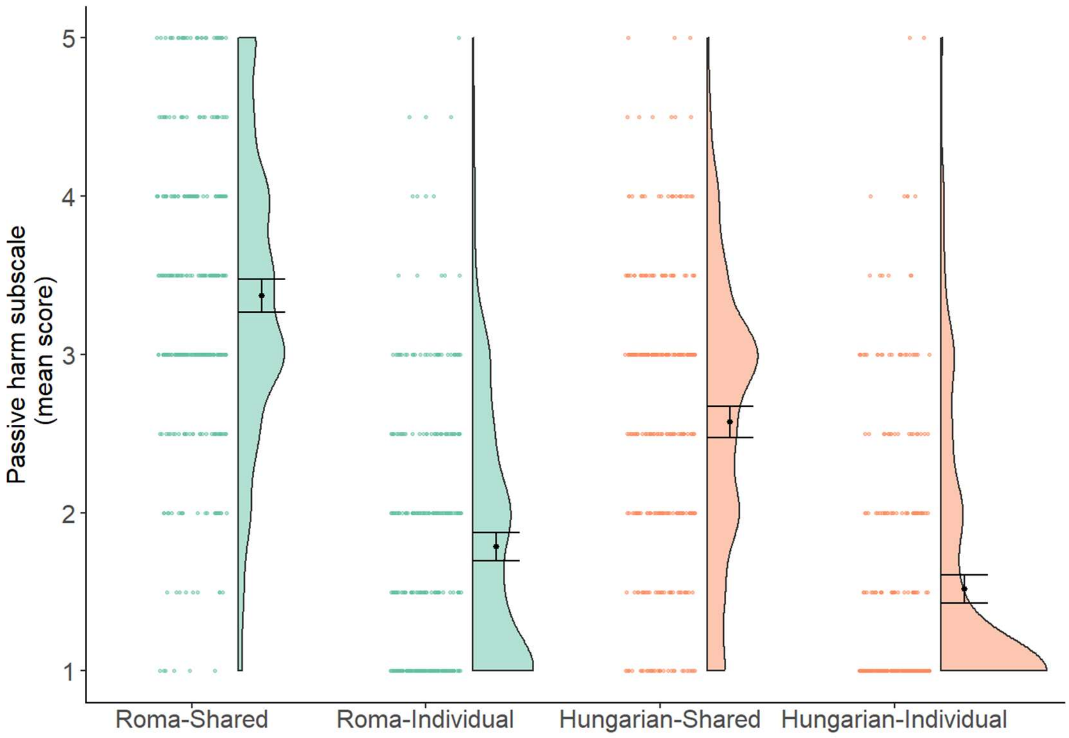

Supplementary Figure 20. Distributions, means and 95% confidence interval of **passive harm** subscale in respective experimental conditions.

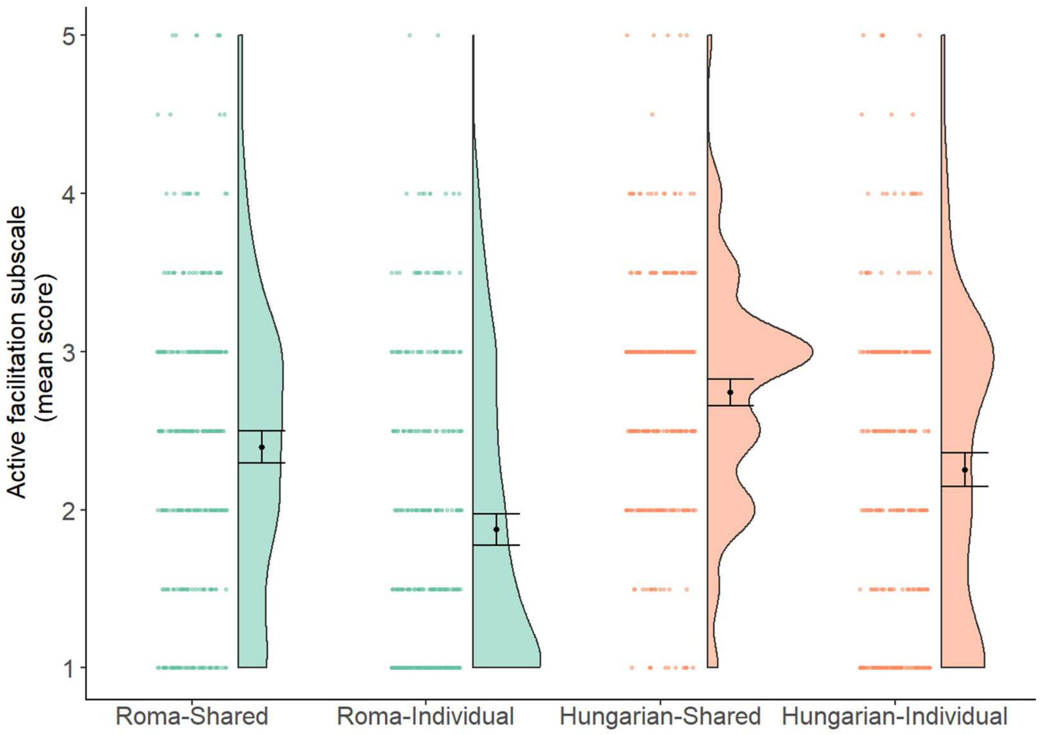

Supplementary Figure 31. Distributions, means and 95% confidence interval of **active facilitation** subscale in respective experimental conditions.

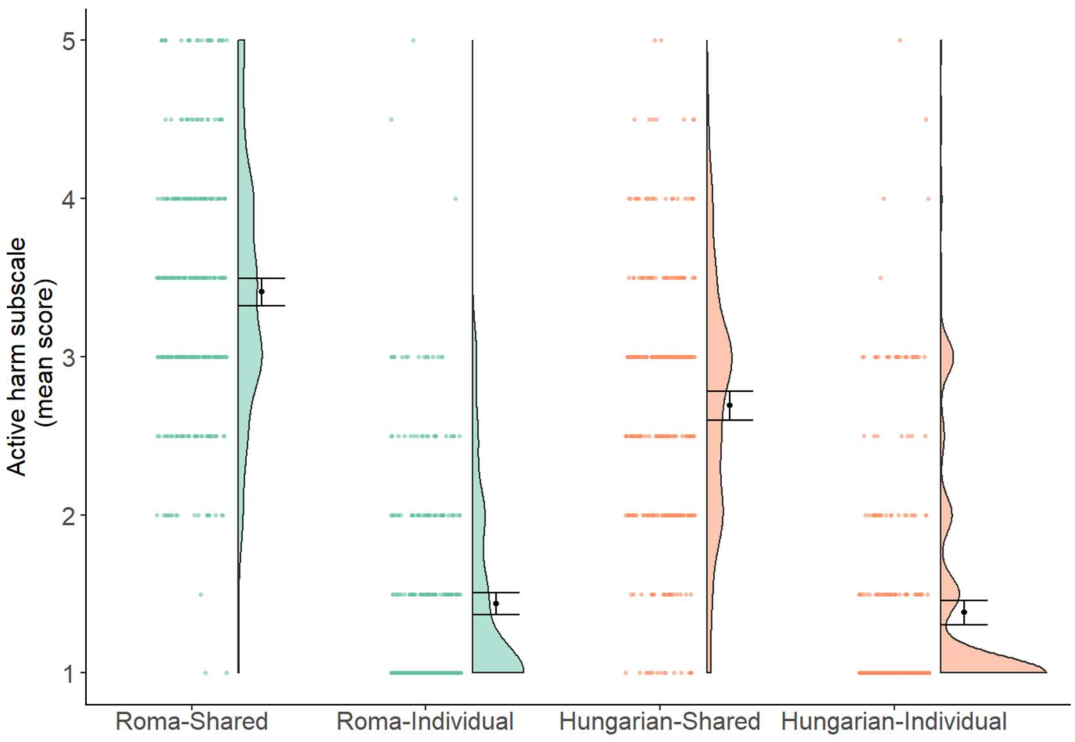

Supplementary Figure 42. Distributions, means and 95% confidence interval of **active harm** subscale in respective experimental conditions.

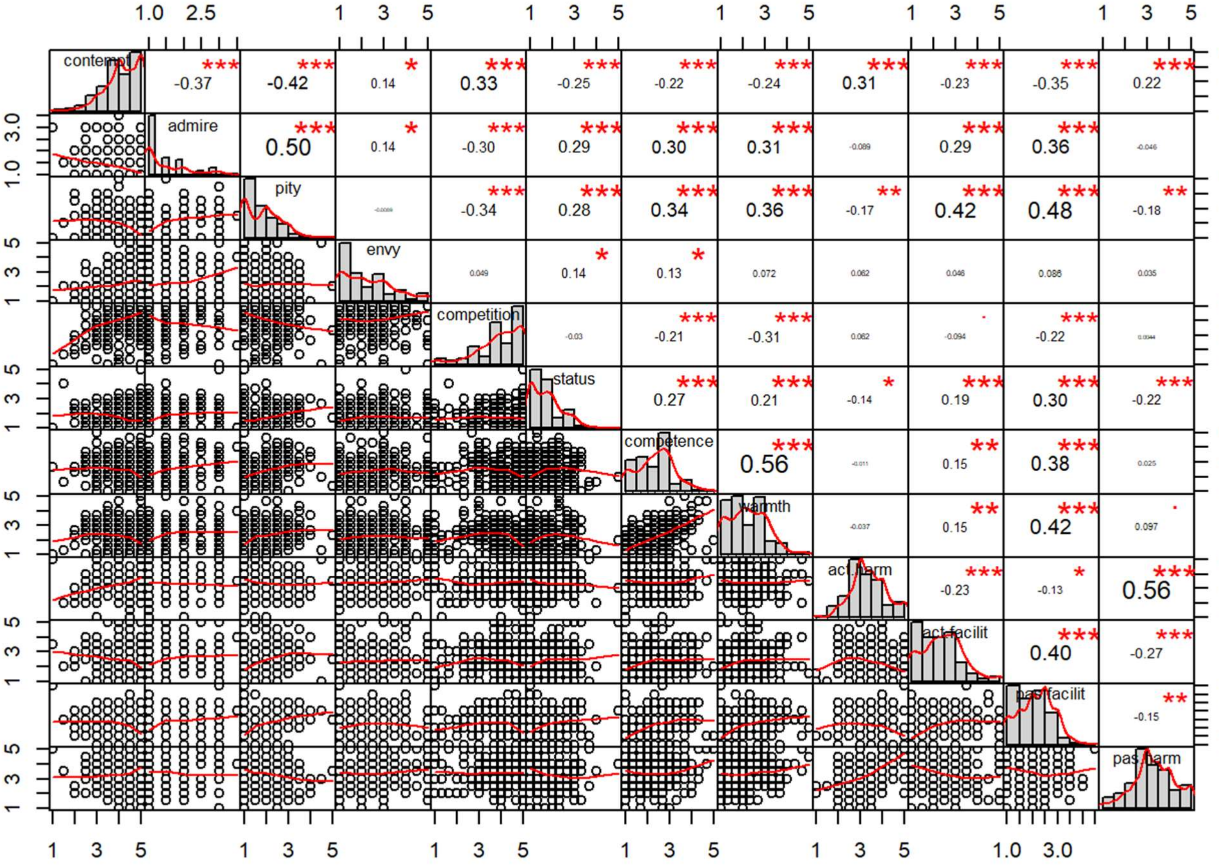

Supplementary Figure 53. Correlations between the BIAS map subscales in the "Roma + shared cultural perspective" experimental condition.

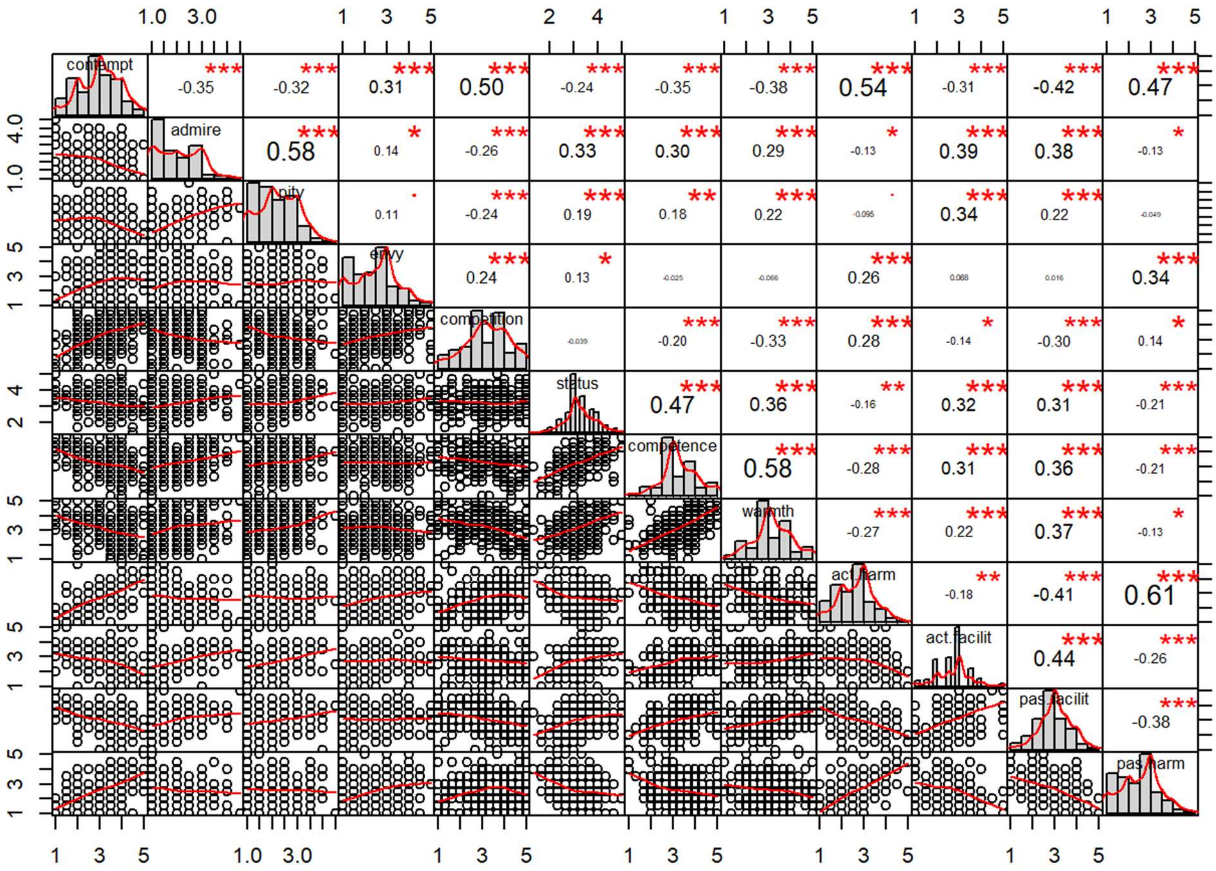

Supplementary Figure 64. Correlations between the BIAS map subscales in the “Hungarian + shared cultural perspective” experimental condition.

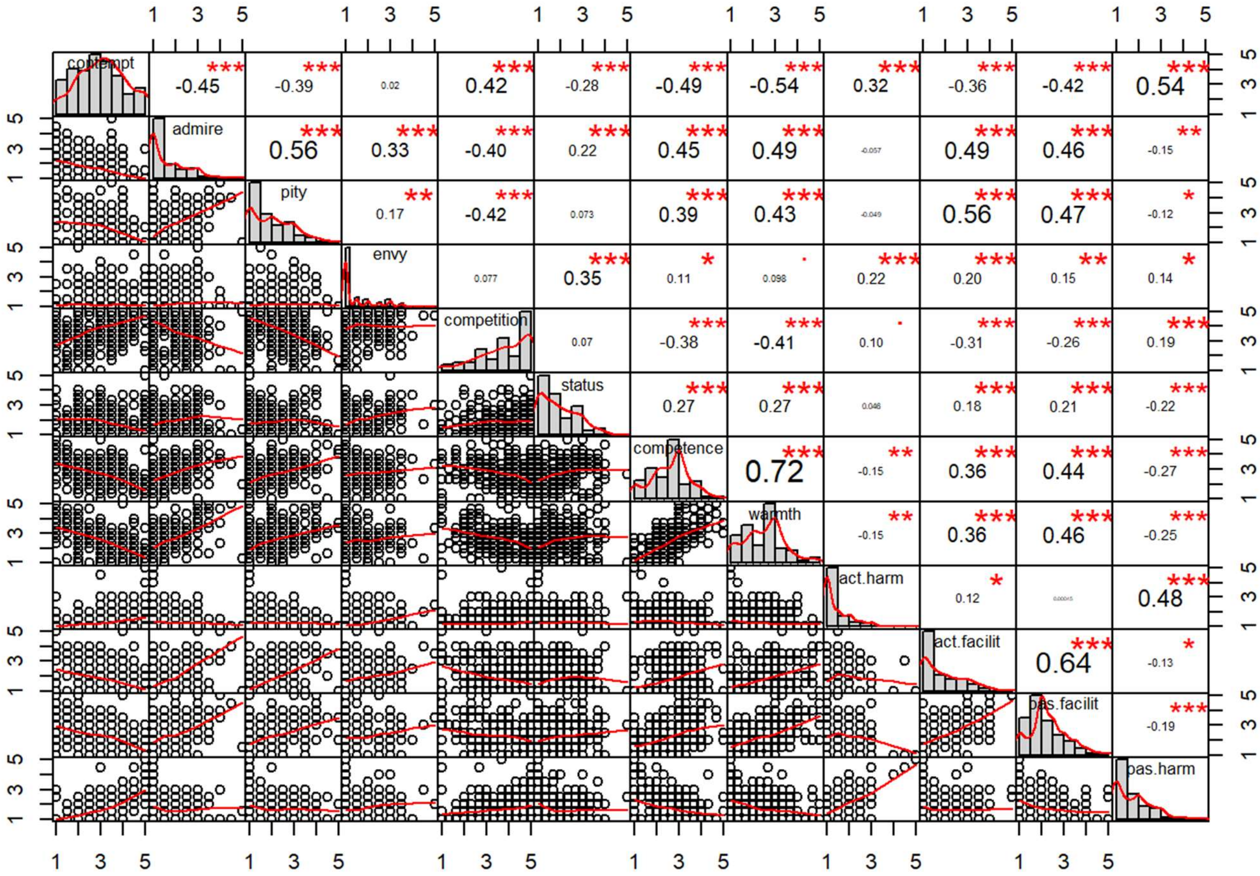

Supplementary Figure 75. Correlations between BIAS map subscales in the “Roma + individual perspective” experimental condition.

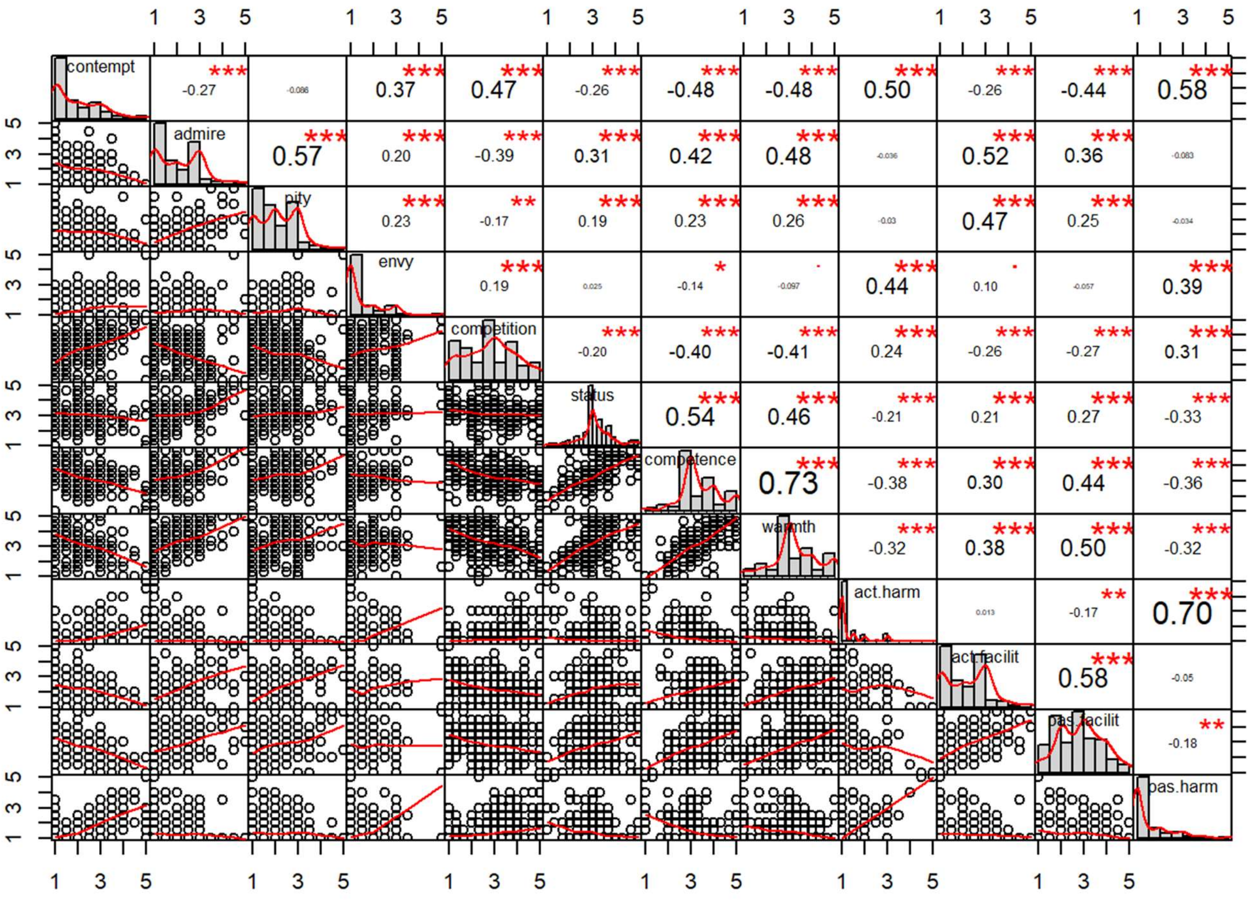

Supplementary Figure 86. Correlations between BIAS map subscales in the "Hungarian + individual perspective" experimental condition.

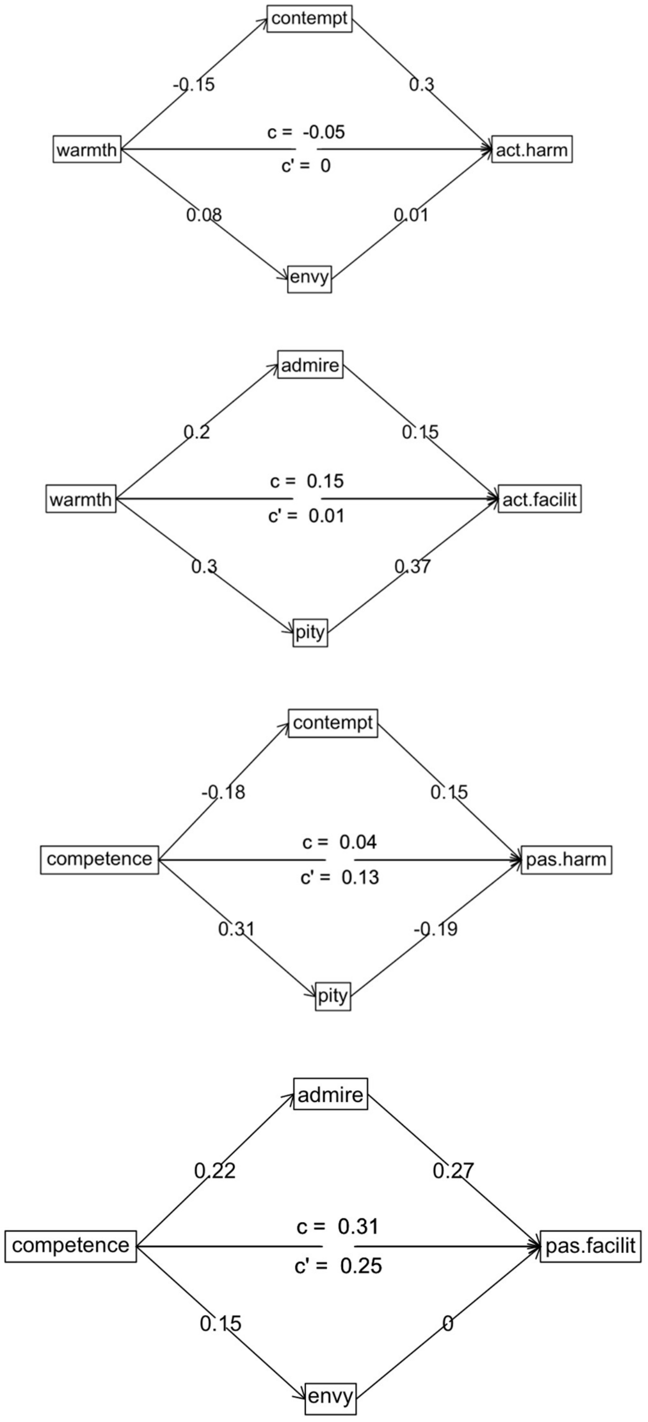

Supplementary Figure 17. BIAS map mediation models of stereotypes predicting emotions predicting behaviors in the “Roma + shared cultural perspective” experimental condition.

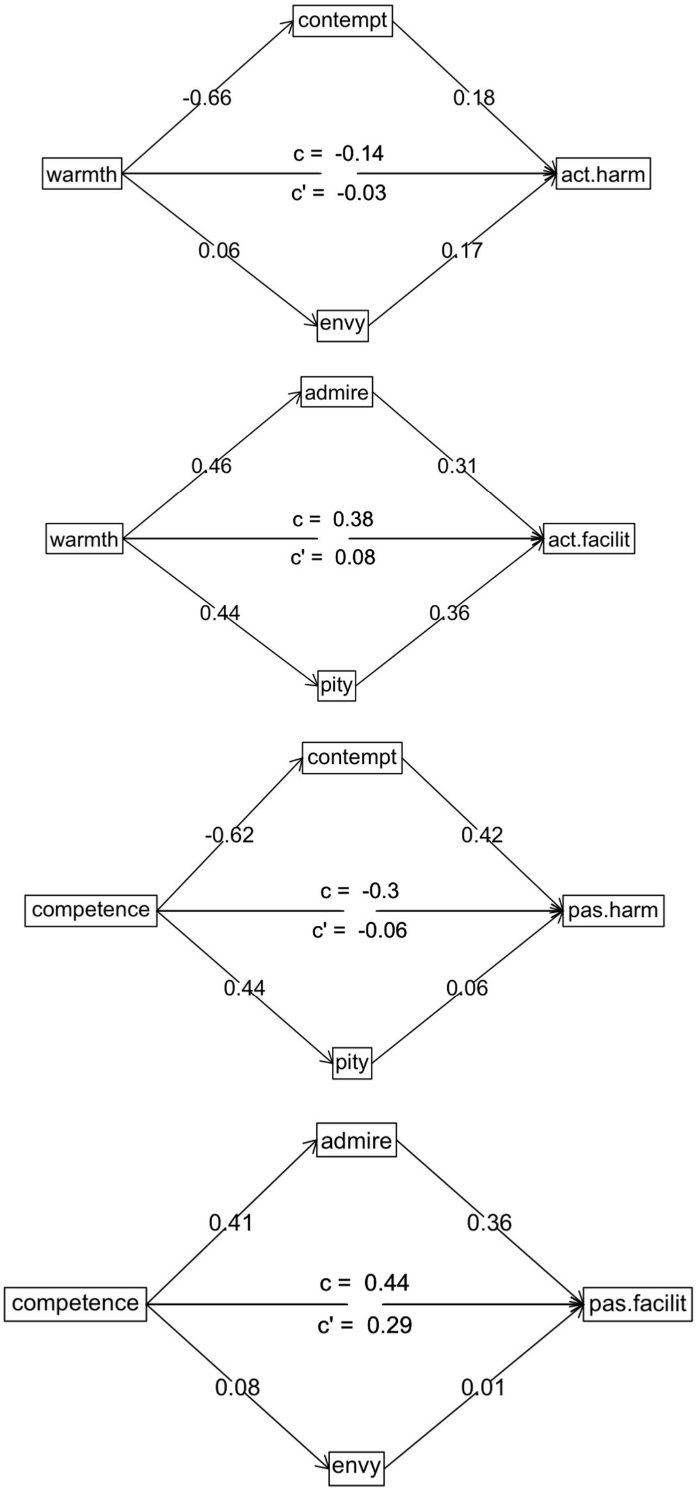

Supplementary Figure 18. BIAS map mediation models of stereotypes predicting emotions predicting behaviors in the “Roma + individual perspective” experimental condition.

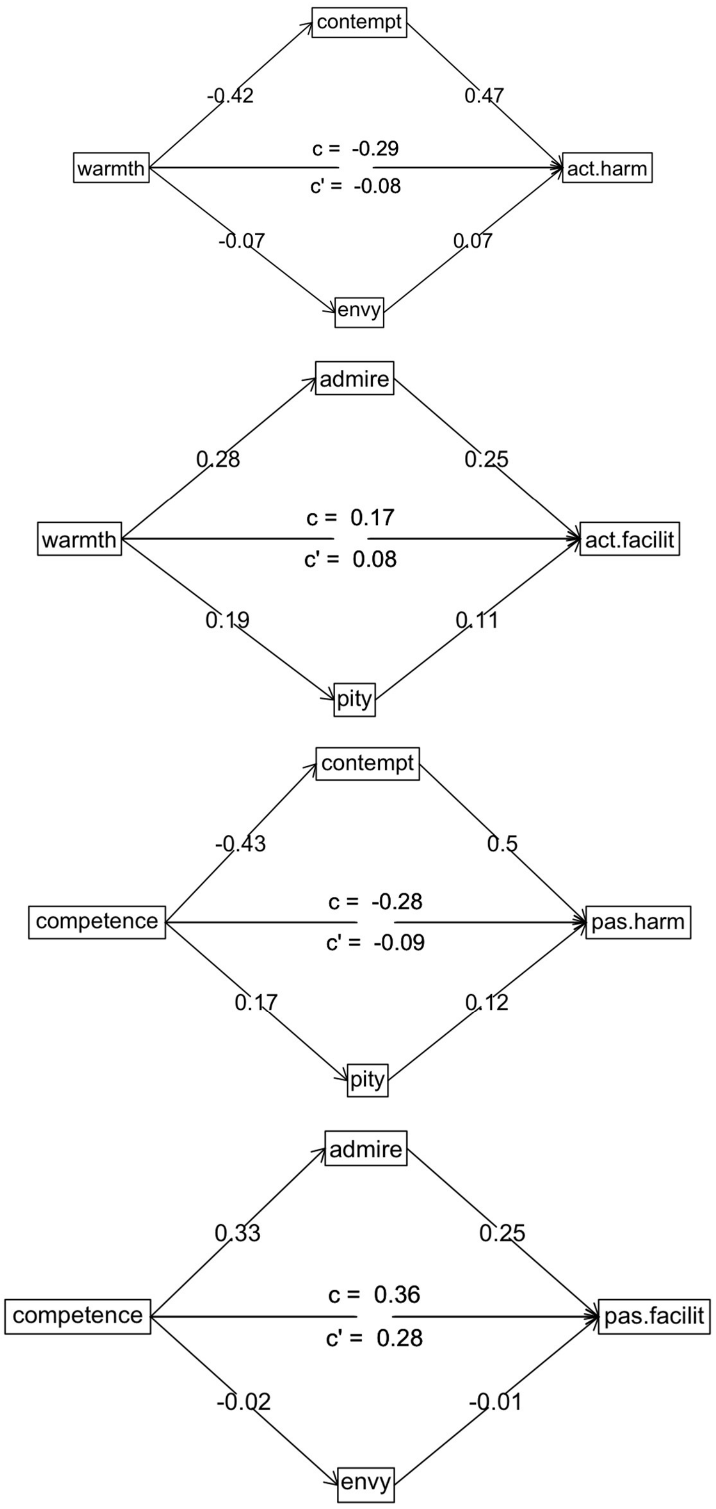

Supplementary Figure 19. BIAS map mediation models of stereotypes predicting emotions predicting behaviors in the "Hungarian + shared cultural perspective" experimental condition.

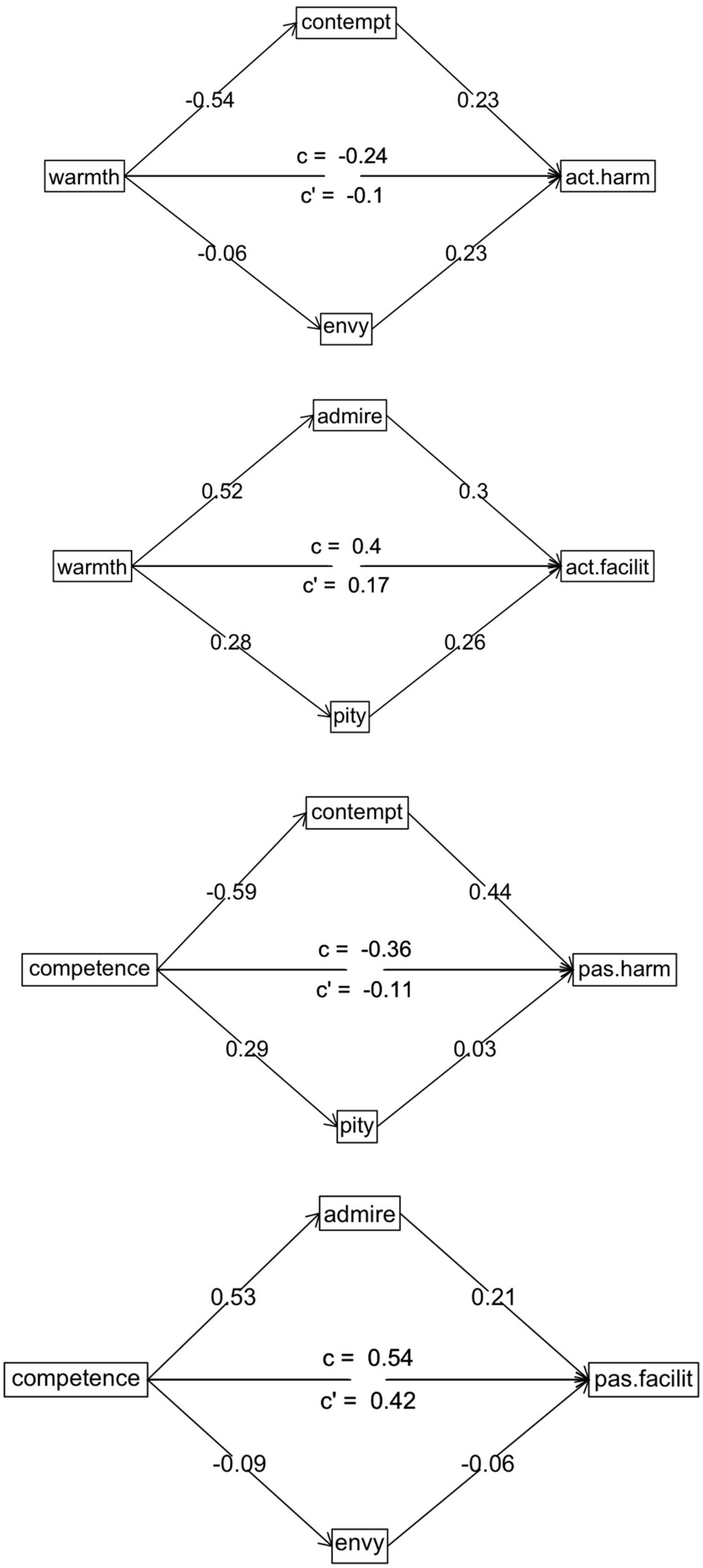

Supplementary Figure 20. BIAS map mediation models of stereotypes predicting emotions predicting behaviors in the “Hungarian + individual perspective” experimental condition.

Supplementary Table 9. *Means and standard deviations of the BIAS map subscales.*

Subscale	Roma + shared cultural (n = 319)		Hungarians + shared cultural (n = 311)		Roma + individual (n = 323)		Hungarians + individual (n = 316)	
	M	*SD*	*M*	*SD*	*M*	*SD*	*M*	*SD*
Competence	2.37	0.84	3.31	0.81	2.63	0.85	3.41	0.87
Warmth	2.22	0.86	3.19	0.86	2.52	0.91	3.28	0.98
Status	1.75	0.7	3.21	0.62	1.93	0.82	3.15	0.74
Competition	3.96	1	3.24	1	3.82	1.11	2.85	1.13
Contempt	4.16	0.77	3.4	0.92	3.5	1.7	1.96	1.3
Admiration	1.43	0.62	2.6	0.83	1.67	0.82	2.14	0.98
Pity	1.87	0.8	2.25	0.78	2.3	0.94	2.2	0.88
Envy	2.29	1.16	2.56	0.97	1.46	0.78	1.5	0.77
Active harm	3.41	0.79	2.69	0.82	1.44	0.65	1.38	0.71
Passive harm	3.37	0.93	2.57	0.9	1.79	0.82	1.52	0.81
Active facilitation	2.4	0.94	2.74	0.74	1.87	0.91	2.25	0.98
Passive facilitation	2.15	0.71	3.4	0.73	2.3	0.86	2.87	0.97

Supplementary Table 2. *Skewness and kurtosis of the BIAS map subscales.*

Subscale	Roma + shared cultural		Hungarians + shared cultural		Roma + individual		Hungarians + individual	
	Skew	Kurtosis	Skew	Kurtosis	Skew	Kurtosis	Skew	Kurtosis
Competence	0.13	-0.26	0.03	0.25	-0.05	-0.23	0.03	0.14
Warmth	0.34	-0.36	-0.02	-0.15	0.24	-0.17	-0.09	-0.18
Status	0.94	0.9	0.15	0.25	0.68	-0.25	0.09	1.19
Competition	-1.08	0.82	-0.28	-0.43	-0.77	-0.27	0.04	-0.84
Contempt	-0.87	0.63	-0.14	-0.47	0.01	-0.68	0.91	0.12
Admiration	1.37	0.88	0.25	-0.93	1.1	0.53	0.42	-0.54
Pity	0.65	0	0.05	-0.72	0.63	-0.34	0.38	-0.2
Envy	0.67	-0.42	0.09	-0.52	1.71	2.2	1.53	1.98
Active harm	0.11	-0.1	0.02	-0.17	1.81	4.7	2.9	4.19
Passive harm	-0.14	-0.28	0.04	-0.44	0.98	0.72	1.64	2.25
Active facilitation	0.38	-0.06	0.01	0.7	0.85	-0.13	0.29	-0.51
Passive facilitation	0.06	-0.47	-0.14	0.34	0.63	0.38	0.09	-0.61

Supplementary Table 3. *Cronbach's alphas for respective experimental conditions for BIAS map subscales and test of differences between alphas for experimental conditions.*

	Competence	Warmth	Status	Competition
Roma + shared cultural	.80	.83	.64	.71
Roma + individual	.84	.85	.74	.80
Hungarian + shared cultural	.87	.86	.70	.80
Hungarian + individual	.91	.91	.83	.84
df	3	3	3	3
χ^2	24.59	19.04	27.45	13.17
p-value	<.001	<.001	<.001	.004

Supplementary Table 4. *Internal consistency of the BIAS map social structure and stereotypes subscales per experimental condition.*

Subscale	Roma + shared cultural		Hungarian + shared cultural	
	Cronbach's α	McDonald's ω	Cronbach's α	McDonald's ω
Competence	.8	.81	.87	.87
Warmth	.83	.83	.86	.86
Status	.64	.67	.69	.7
Competition	.71	.72	.8	.8
	Roma + individual		Hungarian + individual	
	Cronbach's α	McDonald's ω	Cronbach's α	McDonald's ω
Competence	.84	.84	.91	.91
Warmth	.85	.85	.91	.91
Status	.74	.75	.83	.83
Competition	.8	.81	.84	.84

Supplementary Table 5. *Internal consistency of the BIAS map emotions and behavioural tendencies subscales per experimental condition.*

Subscale	Roma + shared cultural		Hungarian + shared cultural	
	Cronbach's α	Spearman-Brown	Cronbach's α	Spearman-Brown
Contempt	.46	.47	.71	.71
Admiration	.52	.53	.68	.68
Pity	.7	.7	.66	.66
Envy	.68	.68	.78	.78
Active facilitation	.65	.65	.6	.6
Active harm	.22	.22	.53	.53
Passive facilitation	.56	.56	.6	.6
Passive harm	.57	.57	.68	.68
	Roma + individual		Hungarian + individual	
	Cronbach's α	Spearman-Brown	Cronbach's α	Spearman-Brown
Contempt	.67	.67	.79	.79
Admiration	.76	.76	.81	.81
Pity	.76	.76	.59	.6
Envy	.7	.7	.81	.81
Active facilitation	.75	.75	.77	.77
Active harm	.5	.54	.78	.78
Passive facilitation	.6	.6	.66	.66
Passive harm	.45	.52	.8	.81

Supplementary Table 6. *Parallel multiple mediation model for active harm.*

Active harm	Roma + shared cultural				
	Coef.	*SE*	*p*	CI low	CI high
Total effect of warmth	-0.0454	0.052	.3842		
Direct effect of warmth	0.0001	0.0509	.999		
Bootstrapped indirect effect of warmth	-0.0469	0.0224		-0.0944	-0.0083
Bootstrapped indirect effect via contempt	-0.0469	0.0215		-0.0925	-0.0108
Bootstrapped indirect effect via envy	0	0.0043		-0.0095	0.009
$R^2 = 0.0856$, $p < .0001$					
	Roma + individual				
	Coef.	*SE*	*p*	CI low	CI high
Total effect of warmth	-0.1433	0.0395	.0003349		
Direct effect of warmth	-0.0332	0.0452	.4622		
Bootstrapped indirect effect of warmth	-0.1097	0.031		-0.1743	-0.052
Bootstrapped indirect effect via contempt	-0.1208	0.0292		-0.1816	-0.0677
Bootstrapped indirect effect via envy	0.0111	0.0088		-0.0033	0.0309
$R^2 = 0.1508$, $p < .0001$					
	Hungarians + shared cultural				
	Coef.	*SE*	*p*	CI low	CI high
Total effect of warmth	-0.2858	0.0518	<.0001		
Direct effect of warmth	-0.0806	0.0476	.0918		
Bootstrapped indirect effect of warmth	-0.2052	0.0386		-0.2844	-0.1325
Bootstrapped indirect effect via contempt	-0.1998	0.0364		-0.2752	-0.1316
Bootstrapped indirect effect via envy	-0.0054	0.0069		-0.0224	0.005
$R^2 = 0.3581$, $p < .0001$					
	Hungarians + individual				
	Coef.	*SE*	*p*	CI low	CI high
Total effect of warmth	-0.2364	0.0385	<.0001		
Direct effect of warmth	-0.0992	0.04	.01372		
Bootstrapped indirect effect of warmth	-0.1381	0.0365		-0.2129	-0.071
Bootstrapped indirect effect via contempt	-0.1251	0.0361		-0.201	-0.0598
Bootstrapped indirect effect via envy	-0.013	0.0116		-0.038	0.0077
$R^2 = 0.3062$, $p < .0001$					

Supplementary Table 7. *Parallel multiple mediation model for active facilitation.*

Active facilitation	Roma + shared cultural				
	Coef.	*SE*	*p*	CI low	CI high
Total effect of warmth	0.1495	0.0609	.0147		
Direct effect of warmth	0.007	0.0613	.909		
Bootstrapped indirect effect of warmth	0.1438	0.0342		0.0819	0.2155
Bootstrapped indirect effect via admiration	0.03	0.0227		-0.0104	0.0787
Bootstrapped indirect effect via pity	0.1137	0.0317		0.0585	0.1808
$R^2 = 0.1387$, $p < .0001$					
	Roma + individual				
	Coef.	*SE*	*p*	CI low	CI high
Total effect of warmth	0.3777	0.0521	<.0001		
Direct effect of warmth	0.0801	0.0526	.1283		
Bootstrapped indirect effect of warmth	0.2995	0.0429		0.2184	0.3861
Bootstrapped indirect effect via admiration	0.1441	0.0421		0.0681	0.2323
Bootstrapped indirect effect via pity	0.1554	0.0326		0.0954	0.2251
$R^2 = 0.3783$, $p < .0001$					
	Hungarians + shared cultural				
	Coef.	*SE*	*p*	CI low	CI high
Total effect of warmth	0.1735	0.0481	.000363		
Direct effect of warmth	0.0818	0.0473	.08493		
Bootstrapped indirect effect of warmth	0.0924	0.0248		0.0481	0.1452
Bootstrapped indirect effect via admiration	0.0707	0.0253		0.0283	0.1254
Bootstrapped indirect effect via pity	0.0217	0.0165		-0.0064	0.059
$R^2 = 0.1586$, $p < .0001$					
	Hungarians + individual				
	Coef.	*SE*	*p*	CI low	CI high
Total effect of warmth	0.4042	0.0513	<.0001		
Direct effect of warmth	0.1721	0.0538	.001524		
Bootstrapped indirect effect of warmth	0.2313	0.0378		0.1593	0.3065
Bootstrapped indirect effect via admiration	0.1575	0.0394		0.0816	0.2367
Bootstrapped indirect effect via pity	0.0738	0.024		0.03	0.1252
$R^2 = 0.3407$, $p < .0001$					

Supplementary Table 8. *Parallel multiple mediation model for passive harm.*

Passive harm	Roma + shared cultural				
	Coef.	*SE*	*p*	CI low	CI high
Total effect of competence	0.0394	0.0628	.5304		
Direct effect of competence	0.1269	0.0652	.05257		
Bootstrapped indirect effect of competence	-0.0878	0.0285		-0.1482	-0.037
Bootstrapped indirect effect via contempt	-0.0285	0.017		-0.0671	-0.0008
Bootstrapped indirect effect via pity	-0.0592	0.0259		-0.1138	-0.013
$R^2 = 0.0519$, $p = 0.0007681$					
	Roma + individual				
	Coef.	*SE*	*p*	CI low	CI high
Total effect of competence	-0.2959	0.0509	<.0001		
Direct effect of competence	-0.0635	0.053	.2325		
Bootstrapped indirect effect of competence	-0.2324	0.0431		-0.3198	-0.3198
Bootstrapped indirect effect via contempt	-0.2615	0.043		-0.3499	-0.1798
Bootstrapped indirect effect via pity	0.0292	0.0193		-0.0073	0.0695
$R^2 = 0.3107$, $p < .0001$					
	Hungarians + shared cultural				
	Coef.	*SE*	*p*	CI low	CI high
Total effect of competence	-0.2809	0.0614	<.0001		
Direct effect of competence	-0.0864	0.0589	.1434		
Bootstrapped indirect effect of competence	-0.1945	0.0418		-0.2806	-0.1171
Bootstrapped indirect effect via contempt	-0.2157	0.0407		-0.3	-0.1414
Bootstrapped indirect effect via pity	0.0212	0.0137		-0.0005	0.0525
$R^2 = 0.2713$, $p < .0001$					
	Hungarians + individual				
	Coef.	*SE*	*p*	CI low	CI high
Total effect of competence	-0.3636	0.0488	<.0001		
Direct effect of competence	-0.1128	0.0495	.02333		
Bootstrapped indirect effect of competence	-0.2515	0.0483		-0.3494	-0.1636
Bootstrapped indirect effect via contempt	-0.2614	0.0467		-0.3558	-0.1759
Bootstrapped indirect effect via pity	0.0099	0.0119		-0.013	0.0341
$R^2 = 0.3867$, $p < .0001$					

Supplementary Table 9. *Parallel multiple mediation model for passive facilitation.*

Passive facilitation	Roma + shared cultural				
	Coef.	*SE*	*p*	CI low	CI high
Total effect of competence	0.3087	0.0441	<.0001		
Direct effect of competence	0.2517	0.0451	<.0001		
Bootstrapped indirect effect of competence	0.0572	0.0187		0.0244	0.097
Bootstrapped indirect effect via admiration	0.0577	0.0187		0.0251	0.0979
Bootstrapped indirect effect via envy	-0.0005	-0.0005		-0.0127	0.0107
$R^2 = 0.1833$, p <.0001					
	Roma + individual				
	Coef.	*SE*	*p*	CI low	CI high
Total effect of competence	0.4441	0.0506	<.0001		
Direct effect of competence	0.2923	0.0525	<.0001		
Bootstrapped indirect effect of competence	0.153	0.0368		0.0863	0.2303
Bootstrapped indirect effect via admiration	0.1518	0.0384		0.0827	0.2325
Bootstrapped indirect effect via envy	0.0012	0.0053		-0.0096	0.0129
$R^2 = 0.2955$, p <.0001					
	Hungarians + shared cultural				
	Coef.	*SE*	*p*	CI low	CI high
Total effect of competence	0.3624	0.0468	<.0001		
Direct effect of competence	0.2815	0.0474	<.0001		
Bootstrapped indirect effect of competence	0.0814	0.0222		0.042	0.1291
Bootstrapped indirect effect via admiration	0.081	0.0219		0.0427	0.1282
Bootstrapped indirect effect via envy	0.0003	0.0034		-0.0062	0.0082
$R^2 = 0.2338$, p <.0001					
	Hungarians + individual				
	Coef.	*SE*	*p*	CI low	CI high
Total effect of competence	0.5402	0.0556	<.0001		
Direct effect of competence	0.4233	0.0638	<.0001		
Bootstrapped indirect effect of competence	0.1164	0.036		0.0466	0.1882
Bootstrapped indirect effect via admiration	0.1097	0.0341		0.0341	0.0341
Bootstrapped indirect effect via envy	0.0067	0.0067		-0.0042	0.0257
$R^2 = 0.262$, p <.0001					
